# Effects of artificial intelligence implementation on efficiency in medical imaging—a systematic literature review and meta-analysis

**DOI:** 10.1038/s41746-024-01248-9

**Published:** 2024-09-30

**Authors:** Katharina Wenderott, Jim Krups, Fiona Zaruchas, Matthias Weigl

**Affiliations:** Institute for Patient Safety, University Hospital Bonn, Bonn, Germany

**Keywords:** Medical imaging, Health services

## Abstract

In healthcare, integration of artificial intelligence (AI) holds strong promise for facilitating clinicians’ work, especially in clinical imaging. We aimed to assess the impact of AI implementation for medical imaging on efficiency in real-world clinical workflows and conducted a systematic review searching six medical databases. Two reviewers double-screened all records. Eligible records were evaluated for methodological quality. The outcomes of interest were workflow adaptation due to AI implementation, changes in time for tasks, and clinician workload. After screening 13,756 records, we identified 48 original studies to be incuded in the review. Thirty-three studies measured time for tasks, with 67% reporting reductions. Yet, three separate meta-analyses of 12 studies did not show significant effects after AI implementation. We identified five different workflows adapting to AI use. Most commonly, AI served as a secondary reader for detection tasks. Alternatively, AI was used as the primary reader for identifying positive cases, resulting in reorganizing worklists or issuing alerts. Only three studies scrutinized workload calculations based on the time saved through AI use. This systematic review and meta-analysis represents an assessment of the efficiency improvements offered by AI applications in real-world clinical imaging, predominantly revealing enhancements across the studies. However, considerable heterogeneity in available studies renders robust inferences regarding overall effectiveness in imaging tasks. Further work is needed on standardized reporting, evaluation of system integration, and real-world data collection to better understand the technological advances of AI in real-world healthcare workflows. Systematic review registration: Prospero ID CRD42022303439, International Registered Report Identifier (IRRID): RR2-10.2196/40485.

## Introduction

With a rising number of patients and limited staff available, the need for changes in healthcare is a pressing issue^1^. Artificial intelligence (AI) technologies promise to alleviate the current burden by taking over routine tasks, such as monitoring patients, documenting care tasks, providing decision support, and prioritizing patients by analyzing clinical data^2,3^. AI-facilitated innovations are claimed to significantly reduce the workload of healthcare professionals^4,5^.

Several medical specialties have already introduced AI into their routine work, particularly in data-intensive domains, such as genomics, pathology, and radiology^4^. In particular, image-based disciplines have seen substantial benefits from the pattern recognition abilities of AI, positioning them at the forefront of AI integration in clinical care^3,6^. AI technologies expedite the processing of an increasing number of medical images, being used for detecting artifacts, malignant cells or other suspicious structures, and optionally for the succeeding prioritization of patients^7–9^.

To successfully adopt AI in everyday clinical practice, different ways for effective workflow integration can be conceived, largely depending on the specific aim, that is, enhancing the quality of diagnosis, providing reinsurance, or reducing human workload^10,11^. Efficiency outcomes related to AI implementation include shorter reading times or a reduced workload of clinicians to meet the growing demand for interpreting an increasing number of images^12–14^. Thus, whether AI fulfills these aims and enables higher efficiency in everyday clinical work remains largely unknown.

Healthcare systems are complex, combining various components and stakeholders that interact with each other^15^. While the success of AI technology implementation highly depends on the setting, processes, and users, current studies largely focus on the technical features and capabilities of AI, not on its actual implementation and consequences in the clinical landscape^2,3,6,16,17^. Therefore, this systematic review aimed to examine the influence of AI technologies on workflow efficiency in medical imaging tasks within real-world clinical care settings to account for effects that stem from the complex and everyday demands in real-world clinical care, all not being existent in experimental and laboratory settings^18^.

## Results

### Study selection

**Fig. 1:**
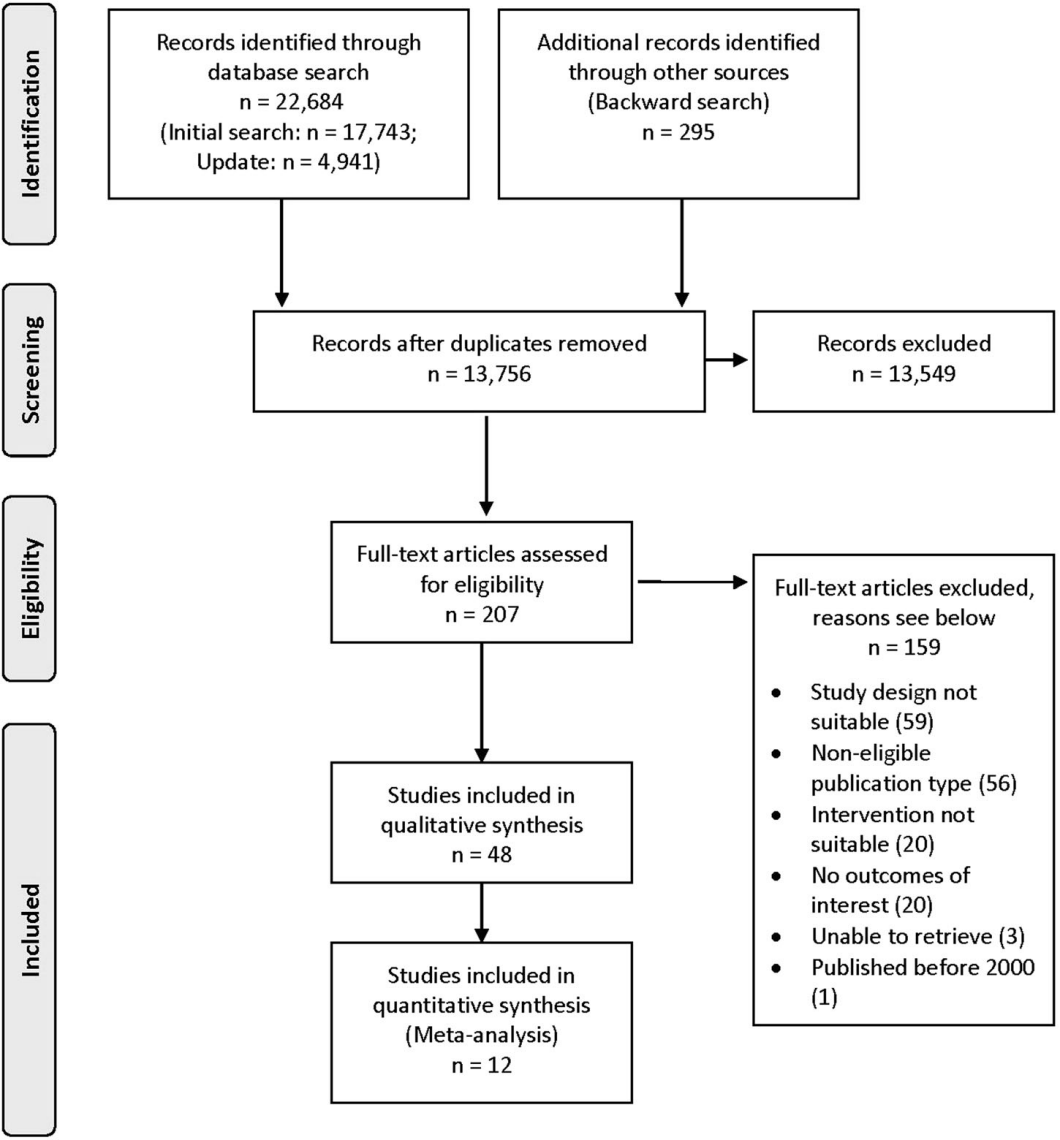
PRISMA flowchart. Visual representation of the search strategy, data screening and selection process of this systematic review.

We identified 22,684 records in databases and an additional 295 articles through backward search. After the removal of duplicates, the 13,756 remaining records were included in the title/abstract screening. Then, 207 full texts were screened, of which 159 were excluded primarily because of inadequate study designs or not focusing on AI for interpreting imaging data (Supplementary Table 1). Finally, 48 studies were included in the review and data extraction. Twelve studies underwent additional meta-analyses. A PRISMA flow chart is presented in Fig. 1.

### Study characteristics

Of the 48 extracted studies, 30 (62.5%) were performed in a single institution, whereas the 18 (37.5%) remaining studies were multicenter studies. One study was published in 2010, another in 2012, and all other included studies were published from 2018 onward. Research was mainly conducted in North America (*n* = 21), Europe (*n* = 12), Asia (*n* = 11), and Australia (*n* = 3). Furthermore, one study was conducted across continents. The included studies were stemming from the medical departments of radiology (*n* = 26), gastroenterology (*n* = 6), oncology (*n* = 4), emergency medicine (*n* = 4), ophthalmology (*n* = 4), human genetics (*n* = 1), nephrology (*n* = 1), neurology (*n* = 1), and pathology (*n* = 1). Most studies used computed tomography (CT) for imaging, followed by X-ray and colonoscopes. The most prominent indications were intracranial hemorrhage, followed by pulmonary embolism, and cancer screening. Table 1 presents the key characteristics of all included studies.

**Table 1:** Key characteristics of included studies

Study	Year	Country	Setting	Medical specialty	Number of professionals	Imaging modality	Number of cases/ patients/ scans
Arbabshirani et al.^7^	2018	USA	Single-Center	Radiology	1	CT	347 patients
Batra et al.^34^	2023	USA	Single-Center	Radiology	32	CT	2501 examinations of 2197 patients
Carlile et al.^80^	2020	USA	Multi-Center	ED	112	X-Ray	1855 scans, survey on 202 scans
Cha et al.^38^	2021	USA	Single-Center	Oncology	18	CT	173 patients
Cheikh et al.^81^	2022	France	Multi-Center	ED	79	CT	7323 examinations
Chen et al.^53^	2022	China	Multi-Center	Radiology	4	CT	85 patients
Conant et al.^28^	2019	USA	Single-Center	Radiology	24	DBT	260 cases
Davis et al.^39^	2022	USA	Multi-Center	Radiology / ED	NI	CT	50,658 cases
Diao et al.^20^	2022	China	Multi-Center	Radiology	7	CT	251 patients
Duron et al.^21^	2021	France	Multi-Center	Radiology / ED	12	X-Ray	600 cases
Elijovich et al.^82^	2022	USA	Multi-Center	Neurology	NI	CT	680 patients
Ginat^83^	2021	USA	Single-Center	Radiology	5	CT	8723 scans
Hassan et al.^40^	2022	USA	Single-Center	Radiology / Neurology	NI	CT	63 patients
Hong et al.^84^	2022	South Korea	Single-Center	Radiology	60	X-Ray	1352 chest radiographs of 1319 patients
Jones et al.^85^	2021	Australia	Multi-Center	Radiology	11	X-Ray	2972 scans of 2665 patients
Kanagasingam et al.^22^	2018	Australia	Single-Center	Ophthalmology	4	Photographs	386 images of 216 patients
Kiljunen et al.^86^	2020	Finland/ Estonia/ Singapore	Multi-Center	Oncology	13	CT	45 scans of 30 patients
Ladabaum et al.^41^	2023	USA	Multi-Center	Gastroenterology	52	Colonoscopy	2329 patients
Levy et al.^87^	2022	Israel	Single-Center	Gastroenterology	30	Colonoscopy	4414 patients
Liu et al.^35^	2022	China	Multi-Center	Ophthalmology	2	OCT	1257 patients
Marwaha et al.^88^	2021	Canada	Single-Center	Human Genetics	15	Photographs	72 patients
Mueller et al.^8^	2022	Denmark	Single-Center	Radiology	2	CT	90 scans
Nehme et al.^29^	2023	USA	Single-Center	Gastroenterology	39	Colonoscopy	1041 patients
O’Neill et al.^89^	2021	USA	Single-Center	Radiology	NI	CT	6696 cases
Oppenheimer et al.^90^	2023	Germany	Single-Center	Radiology	2	X-Ray	1163 exams of 735 patients
Pierce et al.^19^	2021	USA	Single-Center	Radiology	NI	X-Ray	30,847 examinations
Potrezke et al.^54^	2023	USA	Single-Center	Nephrology	12	MRI	170 cases of 161 patients
Quan et al.^91^	2022	USA	Multi-Center	Gastroenterology	6	Colonoscopy	600 patients
Raya-Povedano et al.^36^	2021	Spain	Single-Center	Radiology	5	DM/DBT	15,986 patients
Repici et al.^24^	2020	Italy	Multi-Center	Gastroenterology	6	Colonoscopy	685 patients
Ruamviboonsuk et al.^92^	2022	Thailand	Multi-Center	Ophthalmology	12	Photographs	7651 patients
Sandbank et al.^93^	2022	Israel	Single-Center	Pathology	NI	Microscope	5954 cases
Schmuelling et al.^94^	2021	Switzerland	Single-Center	Radiology	3	CT	1808 scans of 1770 patients
Seyam et al.^95^	2022	Switzerland	Single-Center	Radiology	NI	CT	4450 patients
Sim et al.^96^	2022	Singapore	Single-Center	Radiology	NI	X-Ray	9431 datasets
Strolin et al.^97^	2023	Italy	Single-Center	Oncology	NI	CT	111 patients
Sun et al.^55^	2022	USA	Multi-Center	Radiology	NI	X-Ray	5335 images
Tchou et al.^31^	2010	USA	Single-Center	Radiology	5	DM	267 cases
Tricarico et al.^56^	2022	Italy	Single-Center	Radiology	NI	X-Ray	2942 scans
Vassallo et al.^32^	2019	Italy	Single-Center	Radiology	3	CT	225 patients
Wang et al.^26^	2019	China	Single-Center	Gastroenterology	8	Colonoscopy	1058 patients
Wang et al.^98^	2020	China	Multi-Center	Radiology	2	CT	2120 patients
Wittenberg et al.^33^	2012	Netherlands	Single-Center	Radiology	6	CT	209 patients
Wong et al.^99^	2021	Canada	Multi-Center	Oncology	39	CT	606 radiotherapy plans
Wong et al.^100^	2023	USA	Single-Center	Radiology	17	X-Ray	214 scans
Yacoub et al.^37^	2022	USA	Single-Center	Radiology	3	CT	390 scans
Yang et al.^101^	2022	China	Multi-Center	Ophthalmology	NI	Photographs	1001 patients
Zia et al.^30^	2022	Australia	Single-Center	Radiology	49	CT	1446 scans

*ED* Emergency Department, *CT* Computed Tomography, *DBT* Digital Breast Tomosynthesis, *DM* Digital Mammography, *MRI* Magnetic Resonance Imaging, *OCT* Optical Coherence Tomography.

Concerning the purpose of using AI tools in clinical work, we classified the studies into three main categories. First, five studies (10.4%) described an AI tool used for segmentation tasks (e.g., determining the boundaries or volume of an organ). Second, 25 studies (52.1%) used AI tools to examine detection tasks to identify suspicious cancer nodules or fractures. Third, 18 studies (37.5%) investigated the prioritization of patients according to AI-detected critical features (e.g., reprioritizing the worklist or notifying the treating clinician via an alert).

Regarding the AI tools described in the studies, 34 studies (70.8%) focused on commercially available solutions (Table 2). Only Pierce et al. did not specify which commercially available algorithm was used^19^. Thirteen studies (27.1%) used non-commercially available algorithms, detailed information on these algorithms is provided in Table 3. Different measures were used to evaluate the accuracy of these AI tools, including sensitivity, specificity, positive predictive value (PPV), negative predictive value (NPV), and area under the curve (AUC). Sensitivity and specificity were the most commonly reported measures (see Tables 2 and 3).

**Table 2:** Overview of the commercial AI tools used in the included studies

Source	Clearance	Body part	Purpose	Technology	Study	Sensitivity	Specificity	Processing time
Aidoc Medical, Tel Aviv, Israel/New York, NY, USA	FDA	Head	Prioritization	Convolutional neural network	Davis et al.^39^	95.0%	99.0%	near real-time
					Ginat ^83^	88.4%	96.1%	3 min
					O’Neill et al.^89^	95.0%	99.0%	30–45 sec
					Seyam et al.^95^	87.2%	93.9%	NI
					Zia et al.^30^	85.7%	96.8%	NI
Aidoc Medical, Tel Aviv, Israel	CE, FDA	Chest	Prioritization	Convolutional neural network	Batra et al.^34^	83.3%	97.1%	NI
					Cheikh et al.^81^	92.6%	95.8%	NI
					Schmuelling et al.^94^	79.6%	95.0%	12.6 min
AITEM Solutions, Turin, Italy	NI	Chest	Prioritization	Convolutional neural network	Tricarico et al.^56^	78.2%	64.2%	NI
Annalise AI, Sydney, Australia	Pre-existing regulatory approval	Chest	Detection	Convolutional neural network	Jones et al.^85^	NI	NI	NI
Digital Diagnostics, Coralville, IA, USA	FDA	Eye	Prioritization	Deep learning and rule-based models	Kanagasingam et al.^22^	NI	92.0%	<3 min
EndoVigilant Inc., MD, USA	NI	Colon	Detection	NI	Quan et al.^91^	90.0%	97.0%	30 frames per sec
FDNA Inc., Sunrise, FL, USA	NI	Face	Detection	NI	Marwaha et al.^88^	NI	NI	NI
Gleamer, Paris, France	NI	Whole body	Detection	Convolutional neural network	Duron et al.^21^	79.4% (reader + AI, patient-wise)	93.6% (reader + AI, patient-wise)	NI
					Oppenheimer et al.^90^	86.9%	84.7%	3 min
Hologic, Marlborough, MA, USA	NI	Breast	Detection	NI	Tchou et al.^31^	NI	NI	NI
iCAD, Nashua, NH, USA	NI	Breast	Detection	Convolutional neural network	Conant et al.^28^	85.0% (reader + AI)	69.6% (reader + AI)	NI
Infervision Technology Co., Ltd., Beijing, China	CE, FDA	Chest	Detection	Deep learning	Diao et al.^20^	NI	NI	NI
Limbus AI, Regina, Saskatchewan, Canada	NI	Whole body	Segmentation	Deep learning	Wong et al.^99^	NI	NI	NI
Lunit, Seoul, South Korea	NI	Chest	Detection	Deep learning	Hong et al.^84^	74.8%	99.8%	NI
Medtronic, Minneapolis, MN, USA	FDA	Colon	Detection	NI	Ladabaum et al.^41^	NI	NI	NI
					Levy et al.^87^	NI	NI	NI
					Nehme et al.^29^	NI	NI	NI
					Repici et al.^24^	99.7%	NI	real-time
MVision AI Oy, Helsinki, Finland	CE, FDA	Whole body	Segmentation	Convolutional neural network	Kiljunen et al.^86^	NI	NI	NI
					Strolin et al.^97^	NI	NI	2.3 min
Philipps Healthcare, Best, The Netherlands	NI	Chest	Detection	NI	Wittenberg et al.^33^	96.0%	22.0%	NI
ScreenPoint Medical, Nijmegen, The Netherlands	CE, FDA	Breast	Prioritization	Deep learning	Raya-Povedano et al.^36^	84.1% (reader + AI)	NI	NI
Shanghai Wision AI Co., Ltd., Shanghai, China	NI	Colon	Detection	Deep learning	Wang et al.^26^	94.4% per image	95.9% per image	real-time
Shenzhen SiBright CO. Ltd., Shenzen, China	NIFDC	Eye	Detection	Ensemble of 3 convolutional neural networks	Yang et al.^101^	86.7%	96.1%	24 sec per eye
Siemens Healthcare, Erlangen, Germany	FDA	Chest	Detection	NI	Mueller et al.^8^	NI	NI	NI
					Yacoub et al.^37^	NI	NI	NI
Viz.ai, San Francisco, CA, USA	FDA	Head	Prioritization	NI	Elijovic et al.^82^	81.0%	NI	NI
					Hassan et al.^40^	87.6%	88.5%	NI

*NI* No information, *CE* Conformité Européenne, *FDA* Food and Drug Administration, *NIFDC* National Institutes for Food and Drug Control.

**Table 3:** Non-commercially available AI algorithms

Study	Developers	Body part	Purpose	Technology	Sensitivity	Specificity	Processing time	Notes
Arbabshirani et al.^7^	SD	Head	Prioritization	Convolutional neural network	70.0%	87.0%	2.3 sec	
Carlile et al.^80^	SD	Lung	Detection	Convolutional neural network	82.8%	72.6%	real-time	
Cha et al.^38^	Elguindi et al.^102^	Multiple	Segmentation	Deep learning	NI	NI	NI	
Chen et al.^53^	SD	Head	Detection	Deep learning	33.3% (reader + AI)	91.5% (reader + AI)	NI	
Liu et al.^35^	Wang et al.^103^	Eye	Detection	Deep learning and rule decision models	98.5%	96.2%	21.4 hours	
Potretzke et al.^54^	SD	Kidney	Segmentation	NI	NI	NI	NI	
Ruamviboonsuk et al.^92^	Gulshan et al.^104^	Eye	Detection	Deep learning	91.4%	95.4%	real-time	Validated in Krause et al.^105^, Ruamviboonsuk et al.^106^
Sandbank et al.^93^	SD	Breast	Detection	Multilayered convolutional neural networks	98.1%	96.2%	real-time	
Sim et al.^96^	SD	Lung	Detection	Deep learning	78.8%	97.1%	NI	
Sun et al.^55^	SD	Lung	Detection	NI	67.0%	77.0%	real-time	
Vassallo et al.^32^	Retico et al.^107^	Lung	Detection	NI	85.0% for lesions >3 mm	NI	19 min	Validated in Torres et al.^108^
Wang et al.^98^	SD	Lung	Prioritization	U-Net-based deep learning model	92.3%	85.1%	0.55 min	
Wong et al.^100^	Brown et al.^109^	Chest	Detection	Open-Source framework (SimpleMind)	88.0%	NI	3–4 min	

*NI* No information, *SD* Self-developed by authors, as described in respective publication.

In total only four studies followed a reporting guideline, three studies^20–22^ used Standards for Reporting of Diagnostic Accuracy (STARD) reporting guideline^23^ and Repici et al.^24^ followed the CONSORT guidelines for randomized controlled trials^25^. Only two studies^24,26^ pre-registered their protocol and none of the included studies provided or used an open-source available algorithm.

### Appraisal of methodological quality

**Fig. 2:**
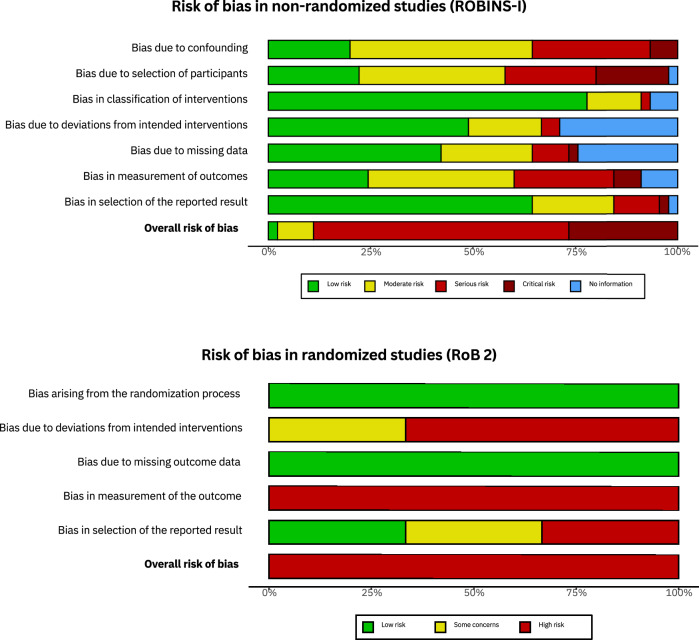
Quality assessment of included articles. Summary plots of the risk of bias assessments via *Risk of Bias in Non-randomized Studies of Interventions tool* (ROBINS-I) for non-randomized studies and the *Cochrane Risk of Bias tool* (Rob 2) for randomized studies.

When assessing the methodological quality of the 45 non-randomized studies only one (2.2%) was rated with an overall “low” risk of bias. Four studies (8.9%) were rated “moderate”, 28 studies (62.2%) were rated “serious”, and 12 studies (26.7%) were rated “critical”. All three randomized studies were appraised with an overall high risk of bias. Summary plots of the risk of bias assessments are shown in Fig. 2, full assessments can be found in Supplementary Figs. 1 and 2. The assessment of the quality of reporting using the *Methodological Index for Non-randomized Studies* (MINORS) is included in Supplementary Figs. 3 and 4. Higher scores indicate higher quality of reporting, with the maximum score being 24 for comparative studies and 16 for non-comparative studies^27^. Comparative studies reported a Median of 9 of 12 criteria with a median overall score of 15 (range: 9–23) and noncomparative studies reported a Median of 7 of 8 checklist items, with a median overall score of 7 (range: 6–14).

### Outcomes

Of all included studies, 33 (68.8%) surveyed the effects of AI implementation on clinicians’ time for task execution. The most frequently reported outcomes included (1) reading time (i.e., time the clinicians required to interpret an image); (2) report turnaround time (i.e., the time from completing the scan until the report is finalized); and (3) total procedure time (i.e., the time needed for colonoscopy)^28–30^. Times were assessed via surveys, recorded by researchers or staff, retrieved via time stamps, or self-recorded. Seventeen studies did not describe how they obtained the reported times.

Regarding our research question, whether AI use improves efficiency, 22 studies (66.6%) reported a reduction in time for task completion due to AI use, with 13 of these studies proving the difference to be statistically significant (see Table 4). Eight studies (24.2%) reported that AI did not reduce the time required for tasks. The remaining three studies (9.1%) chose a design or implementation protocol in which the AI was used after the normal reading, increasing the task time measured by study design^31–33^.

**Table 4:** Outcomes organized by time type measured

Study	Time Type	Assessment Method	Statistical measures	Pre/Without AI	Post/With AI	Absolute Difference (%)	Significance	Workflow Adaptation
Batra et al.^34^	Reading time^a^	Timestamps extracted from EMR and radiologist dictation system	Mean	00:26:30	00:26:18	−00:00:12 (−0.75%)	n.s.	Triage
Cheikh et al.^81^	Reading time^a^	Survey	Mean (SD)	00:14:33 (00:09:05)	00:15:36 (00:09:46)	+00:01:03 ( + 7.22%)	***	Triage
Chen et al.^53^	Reading time	NI	Mean (SD)	00:03:39 (00:00:24)	00:02:56 (00:00:01)	−00:00:43 (−19.77%)	*	NI
Conant et al.^28^	Reading time	NI	Mean (CI)	00:01:04 (00:00:25)	00:00:30 (00:00:12)	−00:00:34 (−52.57%)	**	Second reader, concurrent
Diao et al.^20^	Reading time	Automatically recorded	Mean (SD)	00:04:30 (00:02:24)	00:03:43 (00:02:26)	−00:00:47 (−17.41%)	***	Second reader, sequential
Duron et al.^21^	Reading time	Automatically recorded	Mean	00:01:07	00:00:57	−00:00:10 (−14.93%)	n.s.	Second reader, concurrent
Mueller et al.^8^	Reading time − resident	NI	Mean (SD)	00:06:10 (00:02:49)	00:07:17 (00:02:29)	+00:01:07 ( + 18.11%)	n.s.	Depending on radiologist’s choice
Mueller et al.^8^	Reading time − consultant	NI	Mean (SD)	00:06:06 (00:01:50)	00:06:20 (00:02:01)	+00:00:14 ( + 3.83%)	n.s.	Depending on radiologist’s choice
O’Neill et al.^89^	Reading time^b^	NI	Median (CI)	00:04:50 (00:00:27)	00:06:14 (00:05:28)	+00:01:23 ( + 28.73%)	n.s.	Triage
Schmuelling et al.^94^	Reading time^a^	Timestamps in the clinical information system	Mean (SD)	01:25:30 (04:42:00)	01:18:30 (04:33:00)	−00:07:00 (-8.19%)	n.s.	Triage
Vassallo et al.^32^	Reading time	Recorded by investigator	Mean (SD)	00:04:56 (00:01:20)	00:05:29 (00:01:23)	+00:00:33 ( + 11.15%)	*	Sequential due to study design
Yacoub et al.^37^	Reading time	Self-measured with digital stopwatch	Mean (SD)	00:07:01 (00:02:55)	00:05:28 (00:02:02)	−00:01:33 (−22.09%)	***	Second reader, concurrent
Cha et al.^38^	Contouring time	Self-report	Median (IQR)	00:40:00 (00:43:00)	00:28:00 (00:10:00)	−00:12:00 (−30.00%)	**	First reader
Kiljunen et al.^86^	Contouring time	NI	Mean	00:27:00	00:15:00	−00:12:00 (−44.44%)	NI	First reader
Strolin et al.^97^	Contouring time	NI	Median (Range)	00:25:00 (01:47:00)	00:12:18 (00:46:54)	−00:12:42 (−50.80%)	***	First reader
Potretzke et al.^54^	Segmentation time^c^	..	..	..	..	..	NI	First reader
Tchou et al.^31^	Time to review AI results	Timestamp macro in Excel/ recording by investigator	Mean (SE)	00:01:58 (00:00:04)	..	00:00:23^d^ (00:00:02)	NI	Sequential due to study design
Wittenberg et al.^33^	Time to review AI results	NI	Mean (Range)	00:01:15 (00:01:02)	..	00:00:22^d^ (00:00:18)	NI	Sequential due to study design
Arbabshirani et al.^7^	Time to interpretation^b^	NI	Median (IQR)	08:32:00 (01:51:00)	00:19:00 (00:22:00)	−08:13:00 (−96.29%)	***	Triage
Ginat^83^	Wait time (ED cases)^b^	Automatically recorded	Mean (SD)	01:25:00 (03:14:00)	01:12:00 (02:57:00)	−00:13:00 (−15.29%)	n.s.	Triage
Ginat^83^	Wait time (inpatient cases)^b^	Automatically recorded	Mean (SD)	06:30:00 (06:08:00)	05:52:00 (05:15:00)	−00:38:00 (−9.74%)	**	Triage
Ginat^83^	Wait time (outpatient cases)^b^	Automatically recorded	Mean (SD)	11:14:00 (13:45:00)	01:10:00 (02:21:00)	−10:04:00 (−89.61%)	***	Triage
O’Neill et al.^89^	Wait time^b^	NI	Median (CI)	00:15:45 (00:00:46)	00:12:01 (00:01:55)	−00:03:44 (−23.75%)	***	Triage
Elijovich et al.^82^	Time to notification	Retrospective documentation	Median (IQR)	00:26:00 (00:14:00)	00:07:00 (00:04:00)	−00:19:00 (−73.08%)	***	Triage
Hong et al.^84^	Time to treatment	Retrospectively through analysis of electronic medical records	Mean (SD)	02:30:00 (03:24:00)	01:12:00 (19:30:00)	−01:18:00 (−4.91%)	n.s.	Second reader, concurrent
Batra et al.^34^	Report turnaround time	Timestamps	Mean	00:59:54	00:47:36	−00:12:18 (−20.53%)	***	Triage
Davis et al.^39^	Report turnaround time^a^	NI	Mean (SD)	01:03:30 (01:02:36)	00:52:30 (00:53:55)	−00:11:00 (−17.32%)	**	Triage
Seyam et al.^95^	Report turnaround time^b^	Timestamps extracted from the electronic medical record and PACS	Mean (CI)	01:00:00 (00:17:00)	01:03:00 (00:11:00)	+00:03:00 ( + 5.00%)	NI	Triage
Sim et al.^96^	Report turnaround time	Extracted timestamps from the hospital’s RIS	Mean	00:09:00	00:07:00	−00:02:00 (−22.22%)	NI	Triage
Zia et al.^30^	Report turnaround time^b^	NI	Mean (SD)	01:06:42 (00:41:30)	01:20:00 (01:04:24)	+00:13:18 ( + 19.94%)	*	Second reader, sequential
Schmuelling et al.^94^	ED turnaround time^a^	Timestamps in the clinical information system	Mean (SD)	02:06:00 (01:04:12)	01:59:00 (01:41:00)	−00:07:00 (−5.56%)	n.s.	Triage
Hassan et al.^40^	DIDO time at PSC	NI	Mean (SD)	03:46:42 (04:02:54)	02:04:24 (00:57:36)	−01:42:18 (−45.13%)	*	Triage
Yang et al.^101^	Time for diagnosis	NI	Mean (SD)	00:00:38 (00:00:32)	00:00:24 (00:00:08)	−00:00:14 (−36.84%)	NI	NI
Ladabaum et al.^41^	Withdrawal time	NI	Mean (CI)	00:17:30 (00:01:30)	00:18:00 (00:01:36)	+00:00:30 ( + 2.86%)	n.s.	NI
Nehme et al.^29^	Withdrawal time	NI	Median (IQR)	00:17:00 (00:15:00)	00:18:00 (00:16:00)	+00:01:00 ( + 5.88%)	n.s.	NI
Repici et al.^24^	Withdrawal time	Stopwatch	Mean (SD)	00:07:15 (00:02:29)	00:06:57 (00:01:41)	−00:00:18 (−4.14%)	n.s.	NI
Wang et al.^26^	Withdrawal time	NI	Mean (SD)	00:06:23 (00:01:13)	00:06:53 (00:01:47)	+00:00:30 ( + 7.82%)	***	Second reader, concurrent
Ladabaum et al.^41^	Total procedure time	NI	Mean (CI)	00:26:06 (00:01:36)	00:26:42 (00:01:48)	+00:00:36 ( + 2.30%)	n.s.	NI
Levy et al.^87^	Total procedure time	Recorded by endoscopy nurse	Median (IQR)	00:24:00 (00:17:00)	00:22:00 (00:12:00)	−00:02:00 (−8.33%)	***	NI
Nehme et al.^29^	Total procedure time	NI	Median (IQR)	00:23:00 (00:16:00)	00:24:00 (00:19:00)	+00:01:00 ( + 4.35%)	n.s.	NI
Quan et al.^91^	Total procedure time	NI	Mean (SD)	00:19:30 (00:07:12)	00:21:24 (00:09:06)	+00:01:54 ( + 9.74%)	**	NI
Wang et al.^26^	Total procedure time	NI	Mean (SD)	00:12:06 (00:04:05)	00:12:31 (00:04:23)	+00:00:25 ( + 3.47%)	n.s.	Second reader, concurrent
Carlile et al.^80^	..	..	..	..	..	..	..	Second reader, concurrent
Jones et al.^85^	..	..	..	..	..	..	..	Second reader, concurrent
Kanagasingam et al.^22^	..	..	..	..	..	..	..	Triage + notification
Liu et al.^35^	..	..	..	..	..	..	..	Triage + notification
Marwaha et al.^88^	..	..	..	..	..	..	..	Sequential
Oppenheimer et al.^90^	..	..	..	..	..	..	..	Second reader, sequential
Pierce et al.^19^	..	..	..	..	..	..	..	Triage
Raya-Povedano et al.^36^	..	..	..	..	..	..	..	Gatekeeper
Ruamviboonsuk et al.^92^	..	..	..	..	..	..	..	Gatekeeper
Sandbank et al.^93^	..	..	..	..	..	..	..	Second reader, sequential
Sun et al.^55^	..	..	..	..	..	..	..	Second reader, sequential
Tricarico et al.^56^	..	..	..	..	..	..	..	Triage
Wang et al.^98^	..	..	..	..	..	..	..	Triage + notification
Wong et al.^99^	..	..	..	..	..	..	..	First reader
Wong et al.^100^	..	..	..	..	..	..	..	Second reader, concurrent

*n.s.* Not significant, *AI* Artificial intelligence, *CI* 95% confidence interval, *DIDO* Door-in-door out time, *ED* Emergency department, *EMR* Electronic medical record, *IQR* Interquartile range, *NI* No information, *PACS* Picture archiving and communication system, *PSC* Primary stroke center, *RIS* Radiology information system, *SD* Standard deviation, *SE* Standard error.

Time formats are hh:mm:ss. *p < 0.05, **p < 0.01, ***p < 0.001.

^a^ Time measurements for scans that have been classified positive for pulmonary embolism.

^b^ Time measurements for scans that have been classified positive for intracranial hemorrhage.

^c^ Potretzke et al. reported a reduction in segmentation time but no concrete numbers.

^d^ Additional reading time for AI use.

**Fig. 3:**
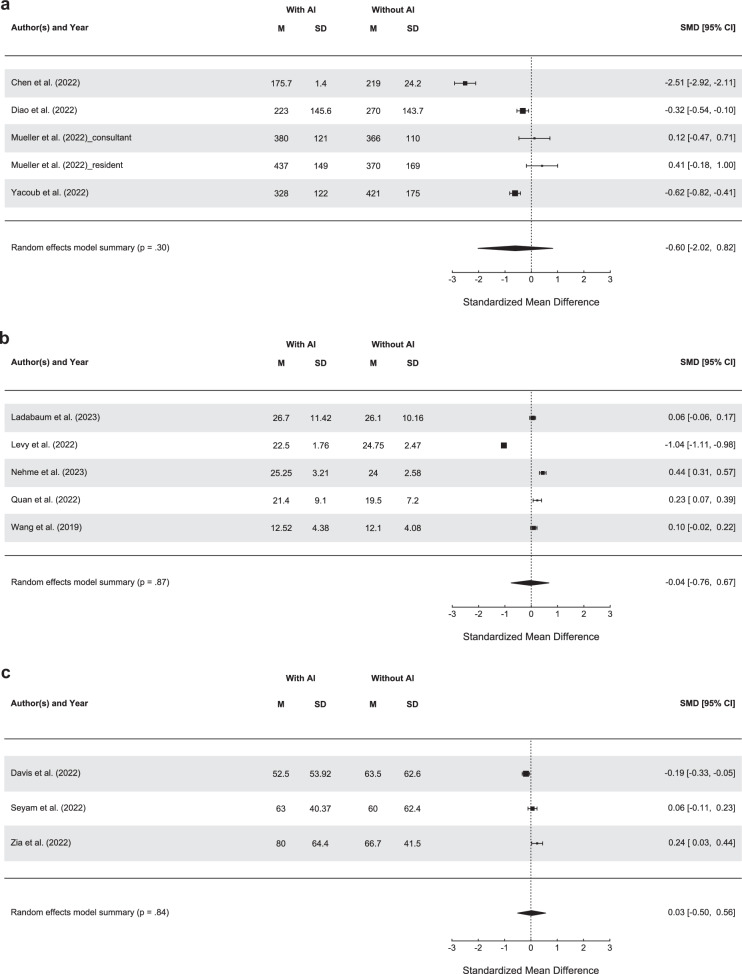
Results of meta-analyses. Graphical display and statistical results of the three meta-analyses: **a** Studies using AI for detection tasks in CT images and reported clinicians’ reading time. **b** Studies using AI to detect polyps during colonoscopy and measured the total procedure time. **c** Studies that used AI for reprioritization and measured the turnaround times for cases flagged positive. All included studies used AIDOC for intracranial hemorrhage detection.

For our meta-analyses, we established clusters with studies deploying similar methods, outcomes, and specific purposes. Concerning studies on detection tasks, we identified two main subgroups: studies using AI for interpreting CT scans (*n* = 7) and those using AI for colonoscopy (*n* = 6). Among studies using AI for interpreting CT images, a meta-analysis was performed for four studies reporting clinicians’ reading times. As shown in Fig. 3a, the reading times for interpreting CT images did not differ between the groups: standardized mean error (SMD): −0.60 (95% confidence interval, −2.02 to 0.82; *p* = 0.30). Furthermore, the studies showed significantly high heterogeneity: Q = 109.72, *p* < 0.01, I^2^ = 96.35%. This heterogeneity may be associated with the different study designs included or the risk of bias ratings, with only one study being rated having a low risk of bias. Furthermore, Mueller et al.^8^ reported no overall reading time but separated it for resident and attending physician, which we included separately in our meta-analysis. Concerning the use of AI for colonoscopy, five studies reported comparable measures. Our random effects meta-analysis showed no significant difference between the groups: SMD: −0.04 (95% CI, −0.76 to 0.67; *p* = 0.87), with significant heterogeneity: Q = 733.51, *p* < 0.01, I^2^ = 99.45% (Fig. 3b). Four of the included studies had a serious risk of bias, whereas one randomized study included was rated with a high risk of bias. Among 11 studies that reported AI use for the prioritization of patients’ scans, four measured the turnaround time. The study by Batra et al.^34^ did not report variance measures and was therefore excluded from the meta-analysis. The remaining three studies used the AI tool Aidoc (Tables 2 and 4) to detect intracranial hemorrhage and reported the turnaround time for cases flagged positive. The meta-analysis showed no significant difference in turnaround time between cases with and without AI use: SMD: 0.03 (95% CI, −0.50 to 0.56; *p* = 0.84), with a significant heterogeneity across studies: Q = 12.31, *p* < 0.01, I^2^ = 83.75% (Fig. 3c). All included studies were non-randomized studies, with two studies being rated with a serious risk of bias and one with a moderate risk of bias.

**Fig. 4:**
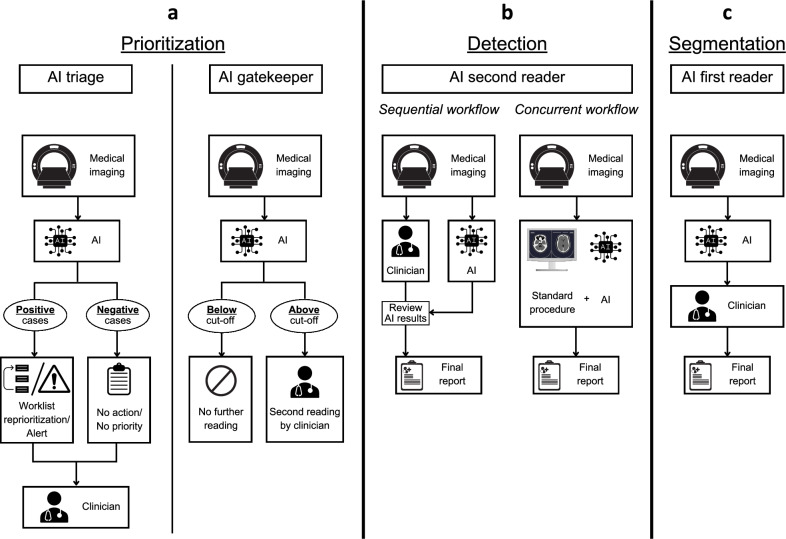
Prototypical workflows after AI implementation. Visual representation of the different workflows when using AI as reported in the included studies: **a** Workflows when using AI for prioritization tasks. **b** Workflow when using AI for detection. **c** Workflow when using AI for segmentation tasks. Figure created with Canva (Canva Pty Ltd, Sydney, Australia).

In total, 37 studies reported details on the actual workflow adaptations due to AI implementation, which we classified into four main variants (depicted exemplarily in Fig. 4). 16 studies (43.2%) used an AI tool as a triage system, i.e., the AI tool reprioritized the worklist or the AI tool sent an alert to the clinician or referred the patient to a specialist for further examination (Fig. 4a: AI triage). In two studies (5.4%), the AI tool acted as a gatekeeper, only referring cases labeled as suspicious to the clinician for further review, while excluding the remaining cases (Fig. 4a: AI gatekeeper). In 13 studies (35.1%), AI tools were used as a second reader for detection tasks in two variants (Fig. 4b: AI second reader). Eight studies reported that the AI tool functioned as a second reader in a concurrent mode, presenting additional information during the task to clinicians (e.g., in colonoscopy studies, where the workflow remained the same as before displaying additional information during the procedure). Five studies described a workflow in which the AI tool was used additionally after the normal detection task, resulting in a sequential second reader workflow. In five segmentation studies (13.5%), the AI tool served as a first reader with the clinician reviewing and then correcting the AI-provided contours (Fig. 4c: AI first reader).

In a single study (2.7%), the type of actual workflow implementation was at the radiologist’s choice. Three studies used a study design with the AI tool as a second reader in a pre-specified reading sequence; therefore, we did not classify them as workflow adaptations. The remaining studies did not provide sufficient information on workflow implementation.

In our initial review protocol, we also aimed to include investigations on clinician workload^14^. Apart from three studies, Liu et al.^35^, Raya-Povedano et al.^36^, and Yacoub et al.^37^, which calculated the saved workload in scans or patients because of AI use, no other study reported AI implementation effects on clinicians’ workload (besides the time for tasks effects, see above). Other reported outcomes included evaluations of the AI performing the task (i.e., satisfaction)^8,38^; frequency of AI use^29,30^; patient outcomes, such as length of stay or in-hospital complications^39,40^; and sensitivity or specificity changes^8,21,24,28,41^.

### Risk of bias across studies

Funnel plots for the studies included in the meta-analyses were created (Supplementary Figs. 5–7). 19 studies declared a relevant conflict of interest and six other studies had potential conflicts of interest, which sum up to more than 50% of the included studies.

Additionally, we ran several sensitivity analyses to evaluate for potential selection bias. We first searched the dblp computer science bibliography, yielding 1159 studies for title and abstract screening. Therein, we achieved perfect interrater reliability (100%). Subsequently, only thirteen studies proceeded to full-text screening, with just one meeting our review criteria. This study by Wismueller & Stockmaster^42^ was also part of our original search. Notably, this study was the only conference publication providing a full paper (refer to Supplementary Table 2).

Moreover, to ensure comprehensive coverage and to detect potentially missed publications due to excluding conference proceedings, we screened 2614 records from IEEE Xplore, MICCAI, and HICSS. Once again, our title and abstract screening demonstrated perfect interrater reliability (100%). However, despite including 31 publications in full-text screening, none met our inclusion criteria upon thorough assessment. Altogether, this additionally searches showed no significant indication for a potential selection bias and potentially missing out key work in other major scientific publication outlets.

Using AMSTAR-2 (A MeaSurement Tool to Assess Systematic Reviews)^43^, we rated the overall confidence in the results as low, mainly due to our decision to combine non-randomized and randomized studies within our meta-analysis (Supplementary Fig. 8).

## Discussion

Given the widespread adoption of AI technologies in clinical work, our systematic review and meta-analysis assesses efficiency effects on routine clinical work in medical imaging. Although most studies reported positive effects, our three meta-analyses with subsets of comparable studies showed no evidence of AI tools reducing the time on imaging tasks. Studies varied substantially in design and measures. This high heterogeneity renders robust inferences. Although nearly 67% of time-related outcome studies have shown a decrease in time with AI use, a noteworthy portion of these studies revealed conflicts of interest, potentially influencing study design or outcome estimation^44^. Our findings emphasize the need for comparable and independent high-quality studies on AI implementation to determine its actual effect on clinical workflows.

Focusing on how AI tools were integrated into the clinical workflow, we discovered diverse adoptions of AI applications in clinical imaging. Some studies have provided brief descriptions that lack adequate details to comprehend the process. Despite predictions of AI potentially supplanting human readers or serving as gatekeepers, with humans primarily reviewing flagged cases to enhance efficiency^10,11^, we noted a limited adoption of AI in this manner across studies. In contrast, most studies reported AI tools as supplementary readers, potentially extending the time taken for interpretation when radiologists must additionally incorporate AI-generated results^18,45^. Another practice involved concurrent reading, which seems beneficial because it guides clinicians’ attention to crucial areas, which potentially improves reading quality and safety without lengthening reading times^45,46^. Regardless of how AI was used, a crucial factor is its alignment with the intended purpose and task^15^.

Although efficiency stands out in the current literature, we were also interested in whether AI affects clinicians’ workload, besides the time measurements, such as number of tasks or cognitive load. We only found three studies on AI’s impact on clinicians’ workload, but no study assessed workload separately (e.g., in terms of cognitive workload changes)^18,35–37^. This gap in research is remarkable since human–technology interaction and human factors assessment will be a success factor for the adoption of AI in healthcare^47,48^.

Our study included a vast variety of AI solutions reported in the publications. The majority was a large number of commercially available AI solutions which mostly had acquired FDA or CE clearance, ensuring safety of use in a medical context^49^. Nevertheless, it is desirable that future studies provide more detailed information about the accuracy of the AI solutions in their use case or processing times, which both can be crucial to AI adoption^50^. Regarding included studies which used non-commercially available algorithms, some of the studies did not specify the origin or source of the algorithm (i.e., developer). Especially with the specific characteristics and potential bias being introduced through the specific algorithm (e.g., for example stemming from a training bias or gaps in the underlying data), it is essential to provide information about the origins and prior validation steps of the algorithm in clinical use^51,52^. Interestingly, only four included studies discussed the possibility of bias in the AI algorithm^53–56^. Open science principles, such as data or code sharing, aid to mitigate the impact of bias. Yet, none of the studies in our review used open-source solutions or provided their algorithm^52^. Additionally, guidelines such as CONSORT-AI or SPIRIT-AI provide recommendations for the reporting of clinical studies using AI solutions^57^, as previous systematic reviews have also identified serious gaps in the reporting on clinical AI solutions^58,59^. Our results corroborate this shortcoming, as none of the studies reporting non-commercial algorithms and only four studies overall followed a reporting guideline. Notwithstanding, for some included studies, AI-specific reporting guidelines were published after their initial publication. Nevertheless, comprehensive and transparent reporting remains insufficient.

With our review, we were able to replicate some of the findings by Yin et al., who provided a first overview on AI solutions in clinical practice, e.g., insufficient reporting in included studies^60^. By providing time for tasks and meta-analyses as well as workflow descriptions our review substantially extends the scope of their review, providing a robust and detailed overview on the efficiency effects of AI solutions. In 2020, Nagendran et al. provided a review comparing AI algorithms for medical imaging and clinicians, concluding that only few prospective studies in clinical settings exist^59^. Our systematic review demonstrated an increase in real-world studies in previous years and provides an up-to-date and comprehensive overview on AI solutions currently used in medical imaging practice. Our study thereby addresses one of the previously mentioned shortcomings, that benefits of the AI algorithm in silico or in retrospective studies might not transfer into clinical benefit^59^. This is also recognized by Han et al.^61^ who evaluated randomized controlled trials evaluating AI in clinical practice and who argued that efficiency outcomes will strongly depend on implementation processes in actual clinical practice.

The complexities of transferring AI solutions from research into practice were explored in a review by Hua et al.^62^ who evaluated the acceptability AI for medical imaging by healthcare professionals. We believe that for AI to unfold its full potential, it is essential to pay thorough attention to the adoption challenges and work system integration in clinical workplaces. Notwithstanding the increasing number of studies on AI use in real-world settings during the last years, many questions on AI implementation and workflow integration remain unanswered. On the one hand, limited consideration prevails on acceptance of AI solutions by professionals^62^. Although studies even discuss the possibility of AI as a teammate in the future^63,64^, most available studies rarely include perceptions of affected clinicians^60^. On the other hand, operational and technical challenges as well as system integration into clinical IT infrastructures are major challenges, as many of the described algorithms are cloud-based. Smooth interoperability between new AI technologies and local clinical information systems as well as existing IT infrastructure is key to efficient clinical workflows^50^. For example, the combination of multimodal data, such as imaging and EHR data, could be beneficial for future decision processes in healthcare^65^.

Our review has several limitations. First, publication bias may have contributed to the high number of positive findings in our study. Second, despite searching multiple databases, selection bias may have occurred, particularly as some clinics implementing AI do not systematically assess or publish their processes in scientific formats^60^. Moreover, we excluded conference publications which could be the source for potential biases. Nevertheless, we ran different sensitivity analyses for publication and selection bias, and did not find evidence for major bias introduced due to our search and identification strategy. Yet, aside from one conference paper, all other conference publications merely provided abstracts or posters, lacking a comprehensive base for the extraction of required details. Third, we focused exclusively on medical imaging tasks to enhance the internal validity of clinical tasks across diverse designs, AI solutions, and workflows. Fourth, the low quality rating of our review on the AMSTAR-2 checklist, which is due to the diverse study designs we included, calling for more comparable high quality studies in this field. Nevertheless, we believe that our review provides a thorough summary of the available studies matching our research question. Finally, our review concentrated solely on efficiency outcomes stemming from the integration of AI into clinical workflows. Yet, the actual impact of AI algorithms on efficiency gains in routine clinical work can be influenced by further, not here specified local factors, e.g., existent IT infrastructure, computational resources, processing times. Next to the testing of the AI solutions under standardized conditions or in randomized controlled trials, which can indicate whether AI solution are suitable for the transfer into routine medical care, careful evaluations of how AI solutions fit into everyday clinical workflow should be expanded, i.e., ideally before implementation. Exploring adoption procedures along with identifying key implementation facilitators and barriers provides valuable insights into successful AI technology use in clinical routines. However, it is important to note that AI implementation can address a spectrum of outcomes, including but not limited to enhancing patient quality and safety, augmenting diagnostic confidence, and improving healthcare staff satisfaction^8^.

In conclusion, our review showed a positive trend toward research on actual AI implementation in medical imaging, with most studies describing efficiency improvements in course of AI technology implementation. We derive important recommendations for future studies on the implementation of AI in clinical settings. The rigorous use of reporting guidelines should be encouraged, as many studies reporting time outcomes did not provide sufficient details on their methods. Providing a protocol or clear depiction of how AI tools modify clinical workflows allows comprehension and comparison between pre- and post-adoption processes while facilitating learning and future implementation practice. Considering the complexity of healthcare systems, understanding the factors contributing to successful AI implementation is invaluable. Our review corroborates the need for comparable evaluations to monitor and quantify efficiency effects of AI in clinical real-world settings. Finally, future research should therefore explore success and potential differences between different AI algorithms in controlled trials as well as in real-world clinical practice settings to inform and guide future implementation processes.

## Methods

### Registration and protocol

Before its initiation, our systematic literature review was registered in a database (PROSPERO, ID: CRD42022303439), and the review protocol was peer-reviewed (International Registered Report Identifier RR2-10.2196/40485)^14^. Our reporting adheres to the Preferred Reporting Items for Systematic Review and Meta-Analysis (PRISMA) statement reporting guidelines (Supplementary Table 3). During the preparation of this work, we used ChatGPT (version GPT-3.5, OpenAI) to optimize the readability and wording of the manuscript. After using this tool, the authors reviewed and edited the content as required and take full responsibility for the content of the publication.

### Search strategy and eligibility criteria

Articles were retrieved through a structured literature search in the following electronic databases: MEDLINE (PubMed), Embase, PsycINFO, Web of Science, IEEE Xplore, and Cochrane Central Register of Controlled Trials. We included original studies on clinical imaging, written in German or English, retrieved in full-text, and published in peer-reviewed journals from the 1st of January 2000 onward, which marked a new area of AI in healthcare with the development of deep learning^14,66^. The first search was performed on July 21st, 2022, and was updated on May 19th, 2023. Furthermore, a snowball search screening of the references of the identified studies was performed to retrieve relevant studies. Dissertations, conference proceedings, and gray literature were excluded. This review encompassed observational and interventional studies, such as randomized controlled trials and nonrandomized studies on interventions (e.g., before–after studies). Only studies that introduced AI to actual real-life clinical workflows were eligible, that is, those not conducted in an experimental setting or in a laboratory. The search strategy followed the PICO framework:

- Population: This review included studies conducted in real-world healthcare facilities, such as hospitals and clinics, using medical imaging and surveying healthcare professionals of varying expertise and qualifications.
- Exposure/interventions: This review encompassed studies that focused on various AI tools for diagnostics and their impact on healthcare professionals’ interaction with the technology across various clinical imaging tasks^67^. We exclusively focused on AI tools that interpret image data for disease diagnosis and screening^5^. For data extraction, we used the following working definition of AI used for clinical diagnostics: “any computer system used to interpret imaging data to make a diagnosis or screen for a disease, a task previously reserved for specialists”^14^.
- Comparators: This review emphasized studies comparing the workflow before AI use with that after AI use or the workflow with AI use with that without AI use, although this was not a mandatory criterion to be included in the review.
- Outcomes: The primary aim of this study was to evaluate how AI solutions impact workflow efficiency in clinical care contexts. Thus, we focused on three outcomes of interest: (1) changes in time required for task completion, (2) workflow adaptation, and (3) workload.

1. *Changes in time* for completion of imaging tasks were considered, focusing on reported quantitative changes attributed to AI usage (e.g., throughput times and review duration).
2. *Workflow adaptation* encompasses changes in the workflow that result from the introduction of new technologies, particularly in the context of AI implementation (i.e., specifying the time and purpose of AI use).
3. *Workload* refers to the demands of tasks on human operators and changes associated with AI implementation (e.g., cognitive demands or task load).

The detailed search strategy following the PICO framework can be found in Supplementary Table 4 and Supplementary Note 1.

### Screening and selection procedure

All retrieved articles were imported into the *Rayyan tool*^68,69^ for title and abstract screening. In the first step, after undergoing a training, two study team members (KW and JK/MW/NG) independently screened the titles and abstracts to establish interrater agreement. In the second step, the full texts of all eligible publications were screened by KW and JK. Any potential conflicts regarding the inclusion of articles were resolved through discussions with a third team member (MW). Reasons for exclusion were documented, as depicted in the flow diagram in Fig. 1^70^.

### Data extraction procedure

Two authors (JK and KW/FZ) extracted the study data and imported them into MS Excel which then went through random checks by a study team member (MW). To establish agreement all reviewers extracted data from the first five studies based on internal data extraction guidelines.

### Study quality appraisal and risk of bias assessment

To evaluate the methodological quality of the included studies, two reviewers (KW and JK) used three established tools. The *Risk of Bias in Non-randomized Studies of Interventions tool* (ROBINS-I) for non-randomized studies and the *Cochrane Risk of Bias tool* (Rob 2) for randomized studies were used^71,72^. To assess the reporting quality of the included studies, the MINORS was used^27^. The MINORS was used instead of the Quality of Reporting of Observational Longitudinal Research checklist^73^, as pre-specified in the review protocol, because this tool was more adaptable to all included studies. Appraisals were finally established through discussion until consensus was achieved.

### Strategy for data synthesis

First, we describe the overall sample and the key information from each included study. Risk of bias assessment evaluations are presented in narrative and tabular formats. Next, where comparable studies were sufficient, a meta-analysis was performed to examine the effects of AI introduction. We used the method of Wan et al.^74^ to estimate the sample mean and standard deviation from the sample size, median, and interquartile range because the reported measures varied across the included studies. Furthermore, we followed the Cochrane Handbook for calculating the standard deviation from the confidence interval (CI)^75^. The *metafor* package in R^76^ was used to quantitatively synthesize data from the retrieved studies. Considering the anticipated heterogeneity of effects, a random-effects model was used to estimate the average effect across studies. Moreover, we used the DerSimonian and Laird method to determine cross-study variance and the Hartung–Knapp method to estimate the variance of the random effect^77,78^. Heterogeneity was assessed using Cochran’s *Q* test^79^ and the I^2^ statistic^75^. In cases where a meta-analysis was not feasible, the results were summarized in narrative form and presented in tabular format.

### Meta-biases

Potential sources of meta-bias, such as publication bias and selective reporting across studies, were considered. Funnel plots were created for the studies included in the meta-analyses.

To assess whether our review is subject to selection bias due to the choice of databases and publication types, we conducted an additional search in the dblp computer science bibliography (with our original search timeframe). As this database did not allow our original search string, the adapted version is found in Supplementary Note 2. Additionally, we performed searches on conference proceedings of the last three years, spanning publications from the January 1st 2020 until May 15th 2023. We surveyed IEEE Xplore and two major conferences not included in the database: International Conference on Medical Image Computing and Computer Assisted Intervention (MICCAI) and Hawaii International Conference on System Sciences (HICSS). We conducted an initial screening of titles and abstracts, with one reviewer (KW) screening all records and JK screening 10% to assess interrater reliability. Full-text assessments for eligibility were then performed by one of the reviewers, respectively (KW or JK). Furthermore, the AMSTAR-2 critical appraisal tool for systematic reviews of randomized and/or non-randomized healthcare intervention studies was used^43^.

## Supplementary information

Supplementary Information

## Data Availability

All data generated or analyzed during this study is available from the corresponding author upon reasonable request.

## Code availability

Code for meta-analyses available via https://github.com/katwend/metaanalyses.
