# Supplementary material for: Effects of artificial intelligence implementation on efficiency in medical imaging—a systematic literature review and meta-analysis
Source: NPJ Digit Med. 2024 Sep 30;7:265. doi: 10.1038/s41746-024-01248-9 (PMC11442995; doi:10.1038/s41746-024-01248-9)
Supplement: Supplementary file 1 — Supplementary Information [file 41746_2024_1248_MOESM1_ESM.pdf]

SUPPLEMENTS

SUPPLEMENTARY TABLE 1: JOURNAL ARTICLES EXCLUDED DURING FULL TEXT-SCREENING

AUTHOR(S)	YEAR	TITLE	DOI / URL	REASON FOR EXCLUSION
Alis et al.	2022	A joint convolutional-recurrent neural network with an attention mechanism for detecting intracranial hemorrhage on noncontrast head CT	10.1038/s41598-022-05872-x	No outcomes of interest
Annarumma et al.	2019	Automated Triaging of Adult Chest Radiographs with Deep Artificial Neural Networks	10.1148/radiol.2018180921	Intervention not suitable
Ardila et al.	2019	End-to-end lung cancer screening with three-dimensional deep learning on low-dose chest computed tomography	10.1038/s41591-019-0447-x	Study design not suitable
Auloge et al.	2019	Augmented reality and artificial intelligence-based navigation during percutaneous vertebroplasty: a pilot randomised clinical trial	https://www.cochranelibrary.com/central/doi/10.1002/central/CN-02119332/full	Intervention not suitable
Bakin et al.	2021	Fast prototyping of a local fuzzy search system for decision support and retraining of hospital staff during pandemic	10.1007/s13755-021-00150-y	Intervention not suitable
Baltruschat et al.	2021	Smart chest X-ray worklist prioritization using artificial intelligence: a clinical workflow simulation	10.1007/s00330-020-07480-7	Study design not suitable
Bange et al.	2021	Implementing automated prognostic models to inform palliative care: more than just the algorithm	10.1136/bmjqs-2021-013510	Non-eligible publication type
Barinov et al.	2019	Impact of Data Presentation on Physician Performance Utilizing Artificial Intelligence-Based Computer-Aided Diagnosis and Decision Support Systems	10.1007/s10278-018-0132-5	Study design not suitable
Bellemo et al.	2019	Artificial intelligence using deep learning to screen for referable and vision-threatening diabetic retinopathy in Africa: a clinical validation study	10.1016/S2589-7500(19)30004-4	No outcomes of interest
Benedikt et al.	2018	Concurrent Computer-Aided Detection Improves Reading Time of Digital Breast Tomosynthesis and Maintains Interpretation Performance in a Multireader Multicase Study	10.2214/AJR.17.18185	Study design not suitable
Beyer et al.	2007	Comparison of sensitivity and reading time for the use of computer-aided detection (CAD) of pulmonary nodules at MDCT as concurrent or second reader	10.1007/s00330-007-0667-1	Study design not suitable
Brown et al.	2019	Integration of Chest CT CAD into the Clinical Workflow and Impact on Radiologist Efficiency	10.1016/j.acra.2018.07.006	Study design not suitable
Buls et al.	2021	Performance of an artificial intelligence tool with real-time clinical workflow integration – Detection of intracranial hemorrhage and pulmonary embolism	10.1016/j.ejmp.2021.03.015	No outcomes of interest
Byun et al.	2021	Evaluation of deep learning-based autosegmentation in breast cancer radiotherapy	10.1186/s13014-021-01923-1	Study design not suitable
Cabitza et al.	2021	Studying human-AI collaboration protocols: the case of the Kasparov's law in radiological double reading	10.1007/s13755-021-00138-8	Study design not suitable
Catchpoole et al.	2022	Application of a Machine Learning-Based Decision Support Tool to Improve an Injury Surveillance System Workflow	10.1055/a-1863-7176	Intervention not suitable

Cavallo et al.	2023	Clinical Implementation of a Combined Artificial Intelligence and Natural Language Processing Quality Assurance Program for Pulmonary Nodule Detection in the Emergency Department Setting	10.1016/j.jacr.2022.12.016	No outcomes of interest
Chen J et al.	2020	Deep learning-based model for detecting 2019 novel coronavirus pneumonia on high-resolution computed tomography	10.1038/s41598-020-76282-0	Study design not suitable
Chen Y, Xing L et al.	2022	Clinical Evaluation of an Auto-Segmentation Tool for Spine SBRT Treatment	10.3389/fonc.2022.842579	Study design not suitable
Chen Y, Vinogradskiy V et al.	2020	Deep Learning-Based Intraprostatic Lesion Segmentation Using Multi-Parametric MRI For Prostate Radiation Therapy	10.1016/j.ijrobp.2020.07.2275	Study design not suitable
Chen N et al.	2023	A fully automatic target detection and quantification strategy based on object detection convolutional neural network YOLOv3 for one-step X-ray image grading	10.1039/d2ay01526a	Study design not suitable
Dahlblom et al.	2023	Breast cancer screening with digital breast tomosynthesis: comparison of different reading strategies implementing artificial intelligence	10.1007/s00330-022-09316-y	No outcomes of interest
De Asis-Cruz et al.	2022	FetalGAN: Automated Segmentation of Fetal Functional Brain MRI Using Deep Generative Adversarial Learning and Multi-Scale 3D U-Net	10.3389/fnins.2022.887634	Study design not suitable
Dembrower et al.	2023	Implications for downstream workload based on calibrating an artificial intelligence detection algorithm by standalone-reader or combined-reader sensitivity matching	10.1117/1.JMI.10.S2.S22405	No outcomes of interest
Ding et al.	2020	Measurement and identification of mental workload during simulated computer tasks with multimodal methods and machine learning	10.1080/00140139.2020.1759699	Intervention not suitable
Do et al.	2020	Augmented Radiologist Workflow Improves Report Value and Saves Time: A Potential Model for Implementation of Artificial Intelligence	10.1016/j.acra.2019.09.014	Intervention not suitable
Dyer et al.	2021	Diagnosis of normal chest radiographs using an autonomous deep-learning algorithm	10.1016/j.crad.2021.01.015	Study design not suitable
Edalati et al.	2022	Implementation and prospective clinical validation of AI-based planning and shimming techniques in cardiac MRI	10.1002/mp.15327	Study design not suitable
Eskreis-Winkler et al.	2021	Using Deep Learning to Improve Nonsystematic Viewing of Breast Cancer on MRI	10.1093/jbi/wbaa102	Study design not suitable
Feng et al.	2019	Deep convolutional neural network for segmentation of thoracic organs-at-risk using cropped 3D images	10.1002/mp.13466	Study design not suitable
Flores-Rodriguez & Cabanillas-Carbonell	2022	Implementation of a Mobile Application based on the Convolutional Neural Network for the Diagnosis of Pneumonia	https://thesai.org/Publications/ViewPaper?Volume=13&Issue=8&Code=IJACSA&SerialNo=53	Study design not suitable
Freer & Ulissey	2001	Screening mammography with computer-aided detection: prospective study of 12,860 patients in a community breast center	10.1148/radiol.2203001282	Unable to retrieve
Gooding et al.	2018	Comparative evaluation of autocontouring in clinical practice: A practical method using the Turing test	10.1002/mp.13200	Study design not suitable
Guermazi et al.	2022	Improving Radiographic Fracture Recognition Performance and Efficiency Using Artificial Intelligence	10.1148/radiol.210937	Study design not suitable
Gulshan et al.	2019	Performance of a Deep-Learning Algorithm vs Manual Grading for Detecting Diabetic Retinopathy in India	10.1001/jamaophthalmol.2019.2004	No outcomes of interest
Herweh et al.	2016	Performance of e-ASPECTS software in comparison to that of stroke physicians on assessing CT scans of acute ischemic stroke patients	10.1177/1747493016632244	Study design not suitable
Heydon et al.	2021	Prospective evaluation of an artificial intelligence-enabled algorithm for automated diabetic retinopathy screening of 30 000 patients	10.1136/bjophthalmol-2020-316594	No outcomes of interest

Hong et al.	2022	Implementation of machine learning in the clinic: challenges and lessons in prospective deployment from the System for High Intensity EvaLuation During Radiation Therapy (SHIELD-RT) randomized controlled study	10.1186/s12859-022-04940-3	Intervention not suitable
Howard et al.	2023	Automated Inline Myocardial Segmentation of Joint T1 and T2 Mapping Using Deep Learning	10.1148/ryai.220050	Study design not suitable
Hwang et al.	2020	Implementation of a Deep Learning-Based Computer-Aided Detection System for the Interpretation of Chest Radiographs in Patients Suspected for COVID-19	10.3348/kjr.2020.0536	Intervention not suitable
Johny & Madhusoodanan	2021	Edge Computing Using Embedded Webserver with Mobile Device for Diagnosis and Prediction of Metastasis in Histopathological Images	10.1007/s44196-021-00040-x	Intervention not suitable
Jung et al.	2014	Who could benefit the most from using a computer-aided detection system in full-field digital mammography?	10.1186/1477-7819-12-168	Study design not suitable
Kau et al.	2022	FDA-approved deep learning software application versus radiologists with different levels of expertise: detection of intracranial hemorrhage in a retrospective single-center study	10.1007/s00234-021-02874-w	No outcomes of interest
Kiser et al.	2020	Novel Autosegmentation Spatial Similarity Metrics Capture the Time Required to Correct Segmentations Better than Traditional Metrics in a Thoracic Cavity Segmentation Workflow	10.1101/2020.05.14.20102103	Study design not suitable
Krenzer et al.	2022	Fast machine learning annotation in the medical domain: a semi-automated video annotation tool for gastroenterologists	10.1186/s12938-022-01001-x	Intervention not suitable
Kural et al.	2022	Accurate identification of EEG recordings with interictal epileptiform discharges using a hybrid approach: Artificial intelligence supervised by human experts	10.1111/epi.17206	Intervention not suitable
Ladefoged et al.	2019	Deep Learning Based Attenuation Correction of PET/MRI in Pediatric Brain Tumor Patients: Evaluation in a Clinical Setting	10.3389/fnins.2018.01005	No outcomes of interest
Lee et al.	2017	Fully Automated Deep Learning System for Bone Age Assessment	10.1007/s10278-017-9955-8	Study design not suitable
Lehman et al.	2019	Mammographic Breast Density Assessment Using Deep Learning: Clinical Implementation	10.1148/radiol.2018180694	No outcomes of interest
Li et al.	2022	Impact of continuous learning on diagnostic breast MRI AI: evaluation on an independent clinical dataset	10.1117/1.JMI.9.3.034502	Study design not suitable
Liu et al.	2022	The challenges facing deep learning–based catheter localization for ultrasound guided high-dose-rate prostate brachytherapy	10.1002/mp.15522	Study design not suitable
Lotan et al.	2022	Development and Practical Implementation of a Deep Learning-Based Pipeline for Automated Pre- and Postoperative Glioma Segmentation	10.3174/ajnr.A7363	Study design not suitable
Lu S-L et al.	2021	Randomized multi-reader evaluation of automated detection and segmentation of brain tumors in stereotactic radiosurgery with deep neural networks	10.1093/neuonc/noab071	Study design not suitable
Lu Y & Zhang	2019	Deep neural networks assisted diagnosis for metastatic pelvic lymph nodes	10.1097/DCR.0000000000001415	Study design not suitable
Lucido et al.	2023	Validation of clinical acceptability of deep-learning-based automated segmentation of organs-at-risk for head-and-neck radiotherapy treatment planning	10.3389/fonc.2023.1137803	Study design not suitable
Lustberg et al.	2018	Clinical evaluation of atlas and deep learning based automatic contouring for lung cancer	10.1016/j.radonc.2017.11.012	Study design not suitable
Mathew et al.	1989	Artificial intelligence in the prediction of operative findings in low back surgery	10.3109/02688698909002791	Published before 2000

Matsoukas et al.	2023	AI software detection of large vessel occlusion stroke on CT angiography: a real-world prospective diagnostic test accuracy study	10.1136/neurintsurg-2021-018391	No outcomes of interest
Matsumoto et al.	2013	Computer-aided detection of lung nodules on multidetector CT in concurrent-reader and second-reader modes: a comparative study	10.1016/j.ejrad.2013.02.005	Study design not suitable
McClatchey et al.	2013	Intelligent grid enabled services for neuroimaging analysis	10.1016/j.neucom.2013.01.042	Study design not suitable
Medela et al.	2022	Automatic SCOring of Atopic Dermatitis Using Deep Learning: A Pilot Study	10.1016/j.xjidi.2022.100107	Study design not suitable
Ming et al.	2021	Evaluation of a novel artificial intelligence-based screening system for diabetic retinopathy in community of China: a real-world study	10.1007/s10792-020-01685-x	No outcomes of interest
Miyake et al.	2013	Comparative performance of a primary-reader and second-reader paradigm of computer-aided detection for CT colonography in a low-prevalence screening population	10.1007/s11604-013-0187-7	Study design not suitable
Morgado et al.	2021	Incremental Learning for Dermatological Imaging Modality Classification	10.3390/jimaging7090180	Intervention not suitable
Morozov et al.	2022	Effect of artificial intelligence technologies on the CT scan interpreting time in COVID-19 patients in inpatient setting	10.17116/profmed20222501114	Non-eligible publication type
Morris et al.	2019	Cardiac substructure segmentation with deep learning for improved cardiac sparing	10.1002/mp.13940	Study design not suitable
Mozaffary et al.	2019	Integration of fully automated computer-aided pulmonary nodule detection into CT pulmonary angiography studies in the emergency department: effect on workflow and diagnostic accuracy	10.1007/s10140-019-01707-x	Study design not suitable
Nguyen et al.	2022	Deployment and validation of an AI system for detecting abnormal chest radiographs in clinical settings	10.3389/fdgth.2022.890759	No outcomes of interest
Ni et al.	2020	A deep learning approach to characterize 2019 coronavirus disease (COVID-19) pneumonia in chest CT images	10.1007/s00330-020-07044-9	Study design not suitable
Ong et al.	2021	Integration of a vertebral fracture identification service into a fracture liaison service: a quality improvement project	10.1007/s00198-020-05710-8	Intervention not suitable
Oscanoa et al.	2023	Accelerated two-dimensional phase-contrast for cardiovascular MRI using deep learning-based reconstruction with complex difference estimation	10.1002/mrm.29441 EA SEP 2022	No outcomes of interest
Otaki et al.	2022	Clinical Deployment of Explainable Artificial Intelligence of SPECT for Diagnosis of Coronary Artery Disease	10.1016/j.jcmg.2021.04.030	Study design not suitable
Park et al.	2023	Identification of Active Pulmonary Tuberculosis Among Patients With Positive Interferon-Gamma Release Assay Results: Value of a Deep Learning-based Computer-aided Detection System in Different Scenarios of Implementation	10.1097/RTI.0000000000000691	Unable to retrieve
Quon et al.	2020	Deep Learning for Automated Delineation of Pediatric Cerebral Arteries on Pre-operative Brain Magnetic Resonance Imaging	10.3389/fsurg.2020.517375	Study design not suitable
Rodrigues et al.	2022	Automated Large Artery Occlusion Detection in Stroke: A Single-Center Validation Study of an Artificial Intelligence Algorithm	10.1159/000519125	Study design not suitable
Rodrígues-Ruiz et al.	2019	Detection of Breast Cancer with Mammography: Effect of an Artificial Intelligence Support System	10.1148/radiol.2018181371	Study design not suitable
Ronmark et al.	2022	Effect of digital-enabled multidisciplinary therapy conferences on efficiency and quality of the decision making in prostate cancer care	10.1136/bmjhci-2022-100588	Intervention not suitable
Roser et al.	2021	Evaluation of the implementation of an approved artificial intelligence system for the detection of diabetic retinopathy	10.1055/a-1521-7645	No outcomes of interest

Rueckel at al.	2021	Artificial intelligence assistance improves reporting efficiency of thoracic aortic aneurysm CT follow-up	10.1016/j.ejrad.2020.109424	Study design not suitable
Salwei et al.	2022	Usability of a Human Factors-based Clinical Decision Support in the Emergency Department: Lessons Learned for Design and Implementation	10.1177/00187208221078625	Intervention not suitable
Savenije et al.	2020	Clinical implementation of MRI-based organs-at-risk auto-segmentation with convolutional networks for prostate radiotherapy	10.1186/s13014-020-01528-0	No outcomes of interest
Schlaeger et al.	2023	Implementation of GAN-Based, Synthetic T2-Weighted Fat Saturated Images in the Routine Radiological Workflow Improves Spinal Pathology Detection	10.3390/diagnostics13050974	Intervention not suitable
Shaaer et al.	2022	Deep-learning-assisted algorithm for catheter reconstruction during MR-only gynecological interstitial brachytherapy	10.1002/acm2.13494	Study design not suitable
Si et al.	2021	Fully end-to-end deep-learning-based diagnosis of pancreatic tumors	10.7150/thno.52508	Study design not suitable
Silva L et al.	2021	Independent real-world application of a clinical-grade automated prostate cancer detection system	10.1002/path.5662	Study design not suitable
Sollini et al.	2023	The Development of an Intelligent Agent to Detect and Non-Invasively Characterize Lung Lesions on CT Scans: Ready for the “Real World”?	10.3390/cancers15020357	Study design not suitable
Song et al.	2023	Comparative validation of AI and non-AI methods in MRI volumetry to diagnose Parkinsonian syndromes	10.1038/s41598-023-30381-w	Study design not suitable
Steiner et al.	2020	Evaluation of the Use of Combined Artificial Intelligence and Pathologist Assessment to Review and Grade Prostate Biopsies	10.1001/jamanetworkopen. 2020.23267	Study design not suitable
Sung et al.	2021	Added Value of Deep Learning–based Detection System for Multiple Major Findings on Chest Radiographs: A Randomized Crossover Study	10.1148/radiol.2021202818	Study design not suitable
Tam et al.	2021	Augmenting lung cancer diagnosis on chest radiographs: positioning artificial intelligence to improve radiologist performance	10.1016/j.crad.2021.03.021	Study design not suitable
Thainimit et al.	2022	Robotic process automation support in telemedicine: Glaucoma screening usage case	10.1016/j.imu.2022.101001	Intervention not suitable
Thakur et al.	2022	Clinically Deployed Computational Assessment of Multiple Sclerosis Lesions	10.3389/fmed.2022.797586	No outcomes of interest
Tian et al.	2022	Endoscopists' Acceptance on the Implementation of Artificial Intelligence in Gastrointestinal Endoscopy: Development and Case Analysis of a Scale	10.3389/fmed.2022.760634	Study design not suitable
Titano et al.	2018	Automated deep-neural-network surveillance of cranial images for acute neurologic events	10.1038/s41591-018-0147-y	Study design not suitable
Urago et al.	2021	Evaluation of auto-segmentation accuracy of cloud-based artificial intelligence and atlas-based models	10.1186/s13014-021-01896-1	Study design not suitable
Walker et al.	2014	Prospective randomized double-blind study of atlas-based organ-at-risk autosegmentation-assisted radiation planning in head and neck cancer	10.1016/j.radonc.2014.08.028	Study design not suitable
Wallace et al.	2022	Impact of Artificial Intelligence on Miss Rate of Colorectal Neoplasia	10.1053/j.gastro.2022.03.007	No outcomes of interest
White et al.	2022	Pre-deployment assessment of an AI model to assist radiologists in chest X-ray detection and identification of lead-less implanted electronic devices for pre-MRI safety screening: realized implementation needs and proposed operational solutions	10.1117/1.JMI.9.5.054504	Intervention not suitable
Wittenberg et al.	2013	Computed tomography pulmonary angiography in acute pulmonary embolism: the effect of a computer-assisted detection prototype used as a concurrent reader	10.1097/RTI.0b013e 3182870b97	Unable to retrieve

Xue et al.	2022	A multi-feature deep learning system to enhance glaucoma severity diagnosis with high accuracy and fast speed	10.1016/j.jbi.2022.104233	Study design not suitable
Yi et al.	2021	DICOM Image ANalysis and Archive (DIANA): an Open-Source System for Clinical AI Applications	10.1007/s10278-021-00488-5	Intervention not suitable
Yoo et al.	2023	Healthcare Professionals' Expectations of Medical Artificial Intelligence and Strategies for its Clinical Implementation: A Qualitative Study	10.4258/hir.2023.29.1.64	Intervention not suitable
Zabel et al.	2021	Clinical Evaluation of Deep Learning and Atlas-Based Auto-Contouring of Bladder and Rectum for Prostate Radiation Therapy	10.1016/j.prro.2020.05.013	Study design not suitable
Zhang et al.	2020	Artificial intelligence-enabled screening for diabetic retinopathy: a real-world, multicenter and prospective study	10.1136/bmjdrc-2020-001596	No outcomes of interest
Zippelius et al.	2022	Diagnostic accuracy of a novel artificial intelligence system for adenoma detection in daily practice: a prospective nonrandomized comparative study	10.1055/a-1556-5984	Study design not suitable

SUPPLEMENTARY FIGURE 1: RISK OF BIAS IN NON-RANDOMIZED STUDIES (ROBINS-I)

Individual assessments of the included non-randomized studies with the Risk of Bias in Non-randomized Studies of Interventions tool (ROBINS-I).

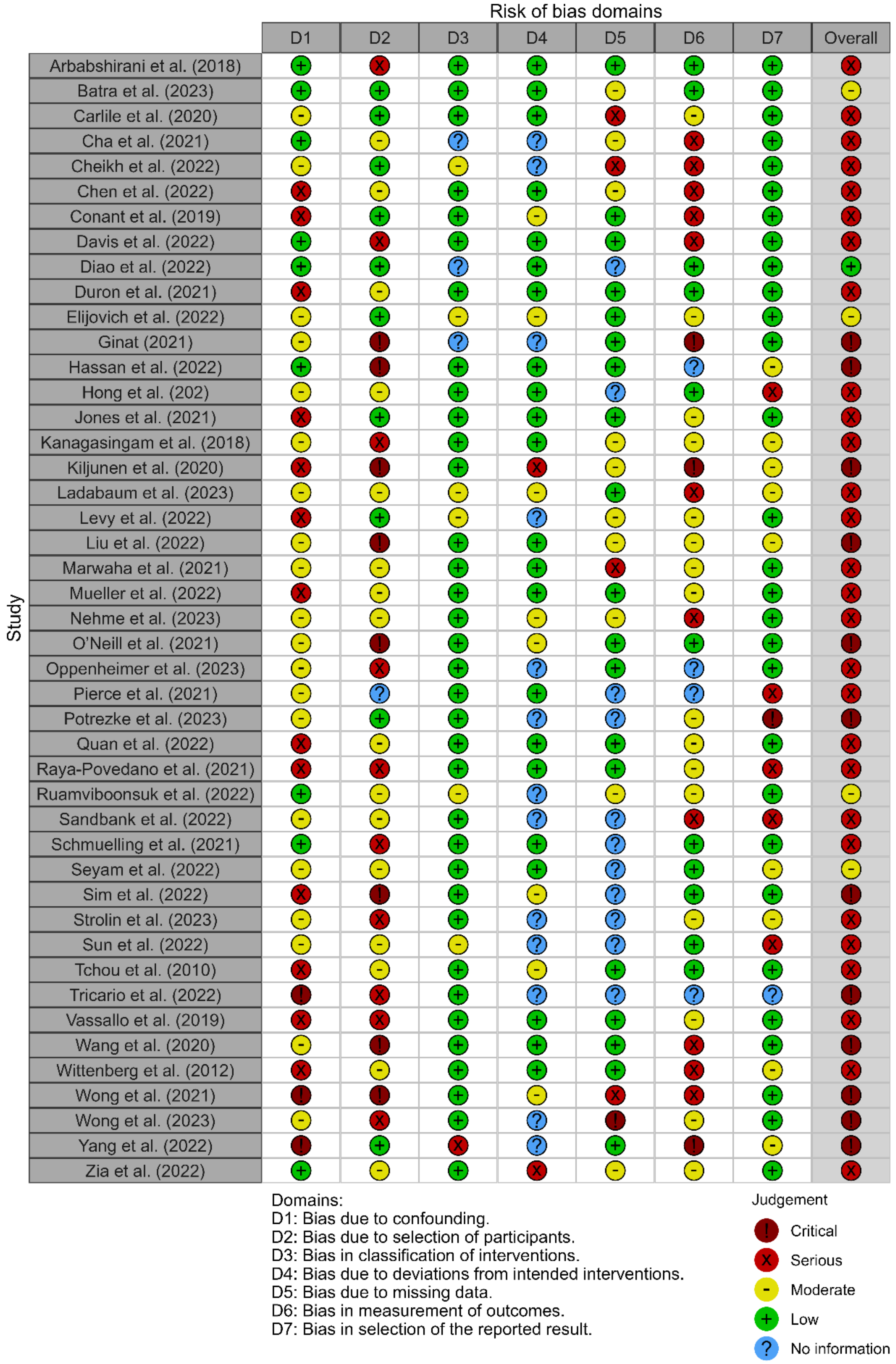

SUPPLEMENTARY FIGURE 2: RISK OF BIAS IN RANDOMIZED STUDIES (ROB-2)

Individual assessments of the included randomized studies with the Cochrane Risk of Bias tool (ROB-2).

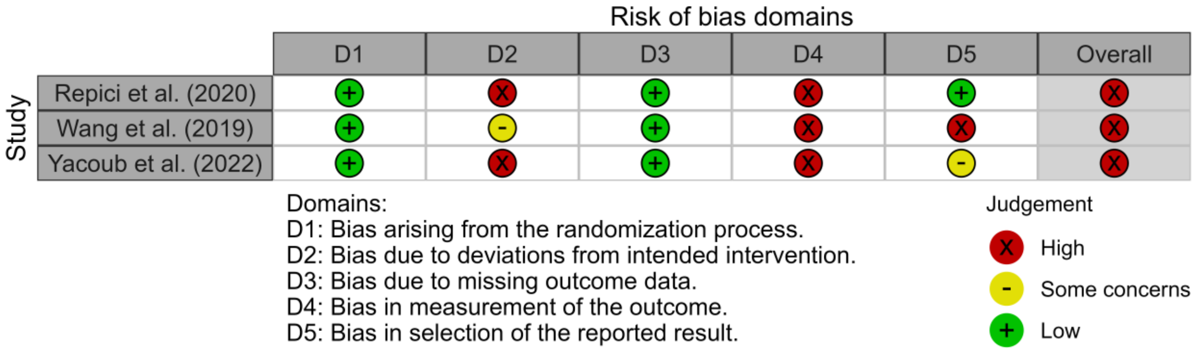

SUPPLEMENTARY FIGURE 3: MINORS FOR COMPARATIVE STUDIES

Individual assessments of the included comparative studies with the Methodological Index for Non-randomized Studies (MINORS).

	Methodological items for non-randomized studies								Additional criteria for comparative studies				
	Clearly stated aim	Inclusion of consecutive patients	Prospective collection of data	Endpoints appropriate to the aim of the study	Unbiased assessment of the study endpoint	Follow-up period appropriate to the aim of the study	Loss to follow up less than 5 %	Prospective calculation of study size	Adequate control group	Contemporary groups	Baseline equivalence of groups	Adequate statistical analysis	Overall (max score 24)
Arbabshirani et al. (2018)	2	2	2	1	2	2	0	0	0	2	0	2	15
Batra et al. (2023)	2	1	0	2	2	2	0	0	2	1	2	2	16
Cha et al. (2021)	2	2	2	2	0	1	1	0	1	0	0	2	13
Cheikh et al. (2022)	2	2	0	2	0	2	1	0	2	0	1	2	14
Chen et al. (2022)	1	1	1	2	1	0	1	0	2	0	2	2	13
Conant et al. (2019)	2	0	1	2	1	1	2	1	1	2	2	2	17
Davis et al. (2022)	2	2	1	2	0	2	2	0	2	0	1	1	15
Diao et al. (2022)	2	2	1	2	2	2	2	0	1	2	0	2	18
Duron et al. (2021)	2	0	1	2	2	2	2	0	2	2	2	2	19
Elijovic et al. (2022)	1	2	0	2	2	2	2	0	1	0	1	2	15
Ginat (2021)	2	1	0	2	1	2	2	0	0	0	0	1	11
Hassan et al. (2021)	2	2	0	2	1	2	2	0	2	0	2	2	17
Hong et al. (2022)	2	2	0	1	2	1	2	1	1	0	1	2	15
Kiljunen et al. (2020)	2	0	0	2	0	0	2	0	1	0	2	0	9
Ladabaum et al. (2023)	2	1	0	2	1	2	2	0	2	1	2	1	16
Levy et al. (2022)	1	2	0	2	1	2	1	0	2	0	2	1	14
Lin et al. (2019)	2	0	0	2	0	2	2	0	2	0	2	2	14
Liu et al. (2020)	2	0	1	1	1	2	2	0	1	1	1	1	13
Marwaha et al. (2021)	2	1	0	1	0	2	0	0	1	1	1	0	9
Mueller et al. (2022)	2	2	2	2	1	1	2	2	1	2	1	2	20
Nehme et al. (2023)	2	1	1	2	1	2	1	0	2	0	2	2	16
O'Neill et al. (2021)	2	2	1	2	2	2	2	0	1	0	1	2	17
Quan et al. (2022)	2	2	1	2	1	2	2	0	2	0	1	2	17
Raya-Povedano et al. (2021)	2	2	0	1	0	2	2	0	1	0	1	1	12
Repici et al. (2020)	1	1	2	2	1	2	2	2	2	2	2	2	21
Schmuelling et al. (2021)	2	1	0	2	2	2	2	0	2	0	1	2	16
Seyam et al. (2022)	2	2	1	2	2	2	2	0	2	1	1	0	17
Sim et al. (2022)	2	0	1	2	2	0	2	0	1	1	0	1	12
Strolin et al. (2023)	1	0	1	2	1	2	2	0	2	0	2	1	14
Tchou et al. (2010)	2	0	1	2	2	2	1	0	0	0	0	2	12
Tricario et al. (2022)	2	0	0	2	1	0	0	0	0	1	2	1	9
Vassallo et al. (2019)	2	2	0	2	1	0	2	0	0	2	2	2	15
Wang et al. (2019)	2	2	2	2	1	2	2	2	2	2	2	2	23
Wang et al. (2020)	1	2	1	2	0	1	2	2	1	1	2	2	17
Yacoub et al. (2022)	2	2	2	2	1	2	0	2	2	2	2	2	21
Yang et al. (2022)	2	2	1	2	0	2	2	1	1	1	0	1	15
Zia et al. (2022)	2	2	1	2	1	2	2	0	2	2	0	0	16

Notes: Green = 2 (reported and adequate), yellow = 1 (reported but inadequate) and red = 0 (not reported)

SUPPLEMENTARY FIGURE 4: MINORS FOR NON-COMPARATIVE STUDIES

Individual assessments of the included non-comparative studies with the Methodological Index for Non-randomized Studies (MINORS).

	Clearly stated aim	Inclusion of consecutive patients	Prospective collection of data	Endpoints appropriate to the aim of the study	Unbiased assessment of the study endpoint	Follow-up period appropriate to the aim of the study	Loss to follow up less than 5 %	Prospective calculation of study size	Overall (max score: 16)
Carlile et al. (2020)	1	1	2	1	1	2	0	0	8
Jones et al. (2021)	2	2	1	2	1	1	1	0	10
Kanagasingam et al. (2018)	1	1	2	1	1	2	1	0	9
Oppenheimer et al. (2023)	2	1	1	2	1	2	2	2	13
Potretzke et al. (2023)	2	0	1	1	1	2	2	0	9
Ruamviboonsuk et al. (2022)	2	1	2	2	1	2	2	2	14
Sandbank et al. (2022)	2	2	1	1	1	2	2	1	12
Sun et al. (2022)	2	2	1	2	2	2	1	2	14
Wittenberg et al. (2012)	1	2	0	1	0	2	2	0	8
Wong et al. (2021)	2	0	1	1	0	2	0	0	6
Wong et al. (2023)	2	0	2	2	1	1	0	0	8

Notes: Green = 2 (reported and adequate), yellow = 1 (reported but inadequate) and red = 0 (not reported)

SUPPLEMENTARY FIGURE 5: FUNNEL PLOT FOR META-ANALYSIS A

Visual investigation of the publication bias for the first meta-analysis on studies using AI for CT detection.

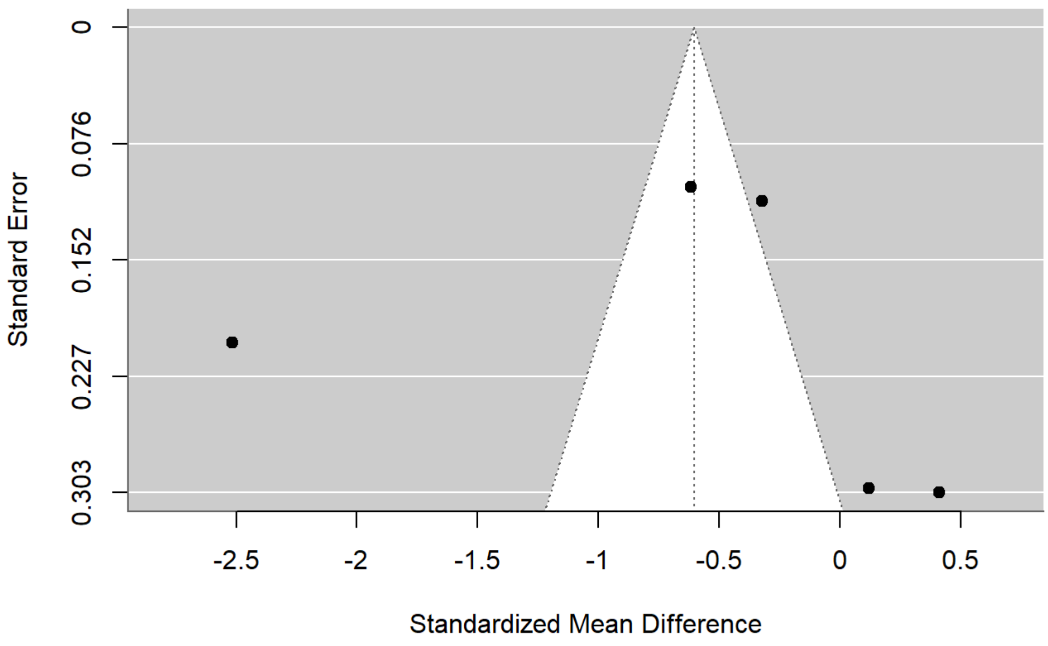

SUPPLEMENTARY FIGURE 6: FUNNEL PLOT FOR META-ANALYSIS B

Visual investigation of the publication bias for the second meta-analysis on studies using AI for endoscopy.

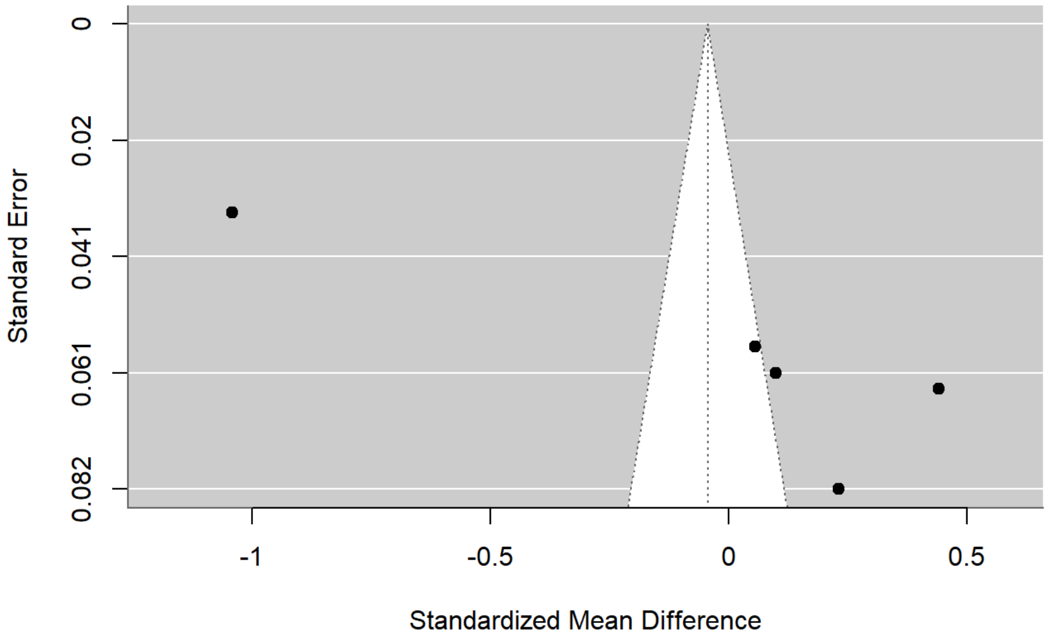

SUPPLEMENTARY FIGURE 7: FUNNEL PLOT FOR META-ANALYSIS C

Visual investigation of the publication bias for the third meta-analysis on studies using AI for re-prioritization of patients.

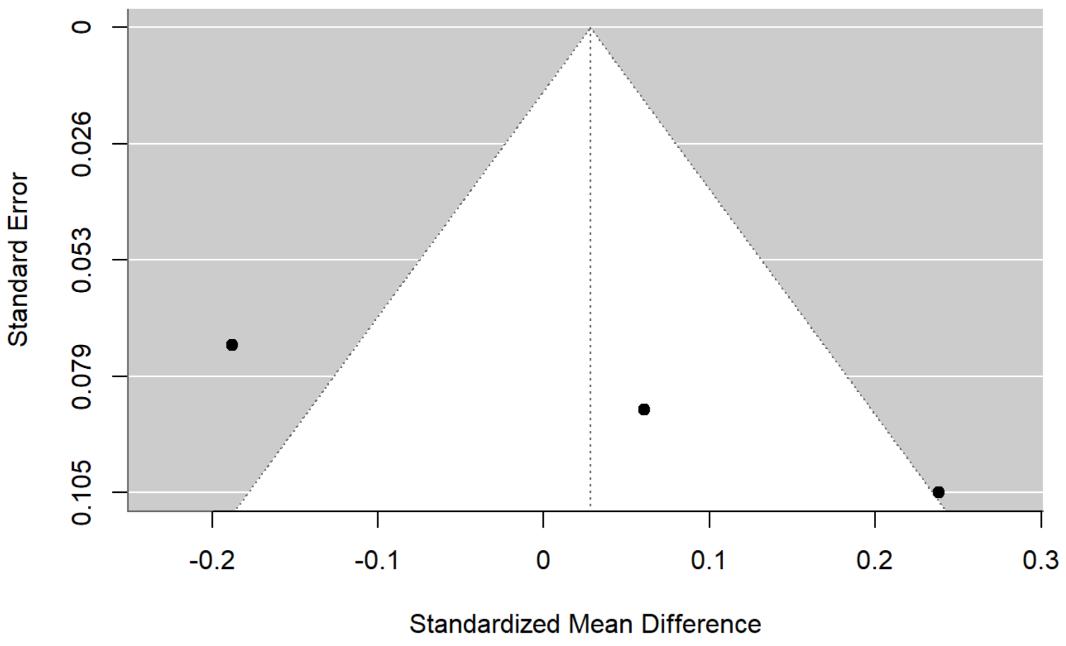

SUPPLEMENTARY TABLE 2: CONFERENCE PAPERS EXCLUDED DURING FULL TEXT-SCREENING

AUTHOR(S)	YEAR	TITLE	DOI / URL	CONFERENCE	PRESENTATION TYPE	AVAILABLE PUBLICATION
Aboian et al.	2022	Development of a workflow efficient PACS based automated brain tumor segmentation and radiomic feature extraction for clinical implementation (N2.003)	10.1212/WNL.98.18_supplement.3146	American Academy of Neurology Annual meeting	Poster	Abstract
Ajmera et al.	2022	Evaluating the effectivity of deploying a deep learning-based algorithm to aid clinicians and radiologists in diagnosing pulmonary nodules on chest radiographs	10.1183/13993003.congress-2022.4308	European Respiratory Society International Congress	NI	Abstract
Almansour et al.	2022	Prospective deployment of a novel deep learning MRI reconstruction for accelerated spine imaging: an analysis of interchangeability, image quality and diagnostic confidence	10.1186/s13244-022-01337-x	European Congress of Radiology	Oral	Abstract
Bangash et al.	2023	Provider Feedback on Implementation of a Genomic Clinical Decision Support for Familial Hypercholesterolemia	10.1016/j.jacl.2021.09.034	NI	Poster	Abstract
Berhanu et al.	2022	Clinical Application of Artificial Intelligence in Multiple Sclerosis: Does Automated Lesion Segmentation Help?	10.1007/s00234-022-03012-w	Annual Meeting of the European Society of Neuroradiology	Poster	Abstract
Chang et al.	2022	Abstract WMP68: Artificial Intelligence For Automated Detection Of Large Vessel Occlusion Using Relative Vessel Density (RAPID LVO): One Year Experience In A Multihospital Integrated Delivery Network	10.1161/str.53.suppl_1.WMP68	International Stroke Conference	Poster	Abstract
Chen K et al.	2022	Integration of Artificial Intelligence into a Telemedicine-Based Diabetic Retinopathy Screening Program	https://iovs.arvojournals.org/article.aspx?articleid=2779657	Association for Research in Vision and Ophthalmology Annual Meeting	NI	Abstract
Coronel et al.	2022	Physician And Staff Attitudes Towards Implementation Of Artificial Intelligence-Assisted Colonoscopy	https://eposters.ddw.org/ddw/2022/ddw-2022/353713/phillip.ge.physician.and.staff.attitudes.towards.implementation.of.artificial.html	Digestive Disease Week	Poster	Abstract & Poster
Dao et al.	2020	IDDF2020-ABS-0165 Accuracy and applicability of the artificial intelligence integrated software in Z-line segmentation	10.1136/gutjnl-2020-IDDF.7	International Digestive Disease Forum (IDDF)	NI	Abstract
Elías Cabot et al.	2022	Evaluation of the performance of artificial intelligence (AI) after one year of use in breast cancer screening practice: is the promise being delivered?	10.1186/s13244-022-01337-x	European Congress of Radiology	Oral	Abstract
Fiorina et al.	2020	222 AI-based strategy enables faster Holter ECG analysis with equivalent clinical accuracy compared to a classical strategy	10.1093/europace/euaa162.374	European Heart Rhythm Association Congress	NI	Abstract
Fraggetta et al.	2019	Implementation of the ContextVision INIFY(TM) tool for the automatic detection of prostatic cancer in a fully digital routine workflow	10.1007/s00428-019-02631-8	European Congress of Pathology	Oral	Abstract
Galoosian et al.	2022	Tu1046: Implementation Of A Machine Learning Algorithm To Measure Adenoma Detection Rates In A Large Health System	https://eposters.ddw.org/ddw/2022/ddw-2022/355211/artin.galoosian.implementation.of.a.machine.learning.algorithm.to.measure.html?f=listing%3D3%2Abrowseby%3D8%2	Digestive Disease Week	Poster	Abstract & Poster

			Asortby%3D2%2Amedia%3D3%2Alabel%3D2 5127			
Goldstein et al.	2022	69-OR: Implementing Autonomous AI in a Federally Qualified Health Center (FQHC) for the Detection of Diabetic Retinopathy Improves Access to Care: A Pre–Post Comparison in the Southeastern U.S.	10.2337/db22-69-OR	American Diabetes Association Scientific Sessions	Oral	Abstract
Greenberg et al.	2022	Image-based identification of HER2 status in H&E-stained breast cancer slides	10.1007/s00428-022-03379-4	European Congress of Pathology	Oral	Abstract
Guthier et al.	2022	PO-1636 Clinical implementation of AI-based contouring workflows from commissioning to automated routine QA	10.1016/S0167-8140(22)03600-3	The European SocieTy for Radiotherapy and Oncology	NI	Abstract
Guthier et al.	2022	Twin AI Algorithms for Quality Control of Auto-Segmentation in Radiation Therapy	https://w4.aapm.org/meetings/	American Association of Physicists in Medicine Annual Meeting	Oral	Abstract
Hassan et al.	2021	Abstract P266: The Implementation of Artificial Intelligence Significantly Reduces Door-In Door-Out Times in Primary Care Center Prior to Transfer	10.1161/str.52.suppl_1.P266	American Stroke Association International Stroke Conference	Poster	Abstract
Hsu et al.	2022	Pilot Implementation of AI Gtvs for SRS zo Brain Metastases	10.1002/mp.15769	American Association of Physicists in Medicine Annual Meeting	Oral	Abstract
Jekel et al.	2022	Nimg-02. Pacs-Integrated Auto-Segmentation Workflow For Brain Metastases Using Nnu-Net	10.1093/neuonc/noac209.622	Society for Neuro-Oncology's Annual Scientific Meeting and Education Day	Poster	Abstract
Lachter et al.	2022	Impacts Of A Novel Ai-Enabled Polyp Detection System: A Prospective Randomized Clinical Trial	10.1002/ueg2.12293	United European Gastroenterology Week	Oral	Abstract
Lerner et al.	2022	OC-0779 MRI-only radiotherapy of gliomas – a prospective clinical study	10.1016/S0167-8140(22)02685-8	The European SocieTy for Radiotherapy and Oncology	NI	Abstract
Levartovsky et al.	2023	P267 Real-world artificial intelligence-aided colonoscopy does not improve adenoma detection rates in patients with Inflammatory Bowel Disease	10.1093/ecco-jcc/jjac190.0397	European Crohn's and Colitis Organisation Congress Copenhagen	Poster	Abstract
Lucido et al.	2022	A Prospective Observational Study of Clinical Acceptability of Deep Learning Model for the Automated Segmentation of Organs at Risk for Head and Neck Radiotherapy Treatment Planning	10.1016/j.ijrobp.2022.07.940	American Society for Radiation Oncology Annual Meeting	Poster	Abstract
Lui et al.	2022	Chest radiograph screening of lung nodules by artificial intelligence algorithm enabling real-time decision support in emergency departments	10.1177/10249079221099636	Asian Conference on Emergency Medicine	Oral	Abstract
Ma et al.	2020	OC-0343 Artificial Intelligence Based Auto-Contouring of CTV for Cervical Cancer	10.1016/S0167-8140(21)00367-4	The European SocieTy for Radiotherapy and Oncology	NI	Abstract
Manjunath et al.	2023	Abstract P5-04-03: Analyzing the performance of Thermalytix, an AI-based breast cancer screening solution, in a community setting	10.1158/1538-7445.SABCS22-P5-04-03	San Antonio Breast Cancer Symposium	Poster	Abstract
Martin et al.	2022	A Systematic And Universal Artificial Intelligence Method For Oropharyngeal Dysphagia Screening: Accuracy Of Machine Learning Is Higher Than Classic Multivariate Analysis	10.1002/ueg2.12294	United European Gastroenterology Week	Poster	Abstract

Misawa et al.	2022	A Prospective Study Of Real-Time Computer-Aided Characterization Of Colorectal Lesions: Diagnostic Performance And Impact On Human Diagnosis	10.1016/j.gie.2022.04.586	Digestive Disease Week	Poster	Abstract
Mohammadzadeh et al.	2022	Detection of Glaucoma Progression on Longitudinal Series of Macular Optical Coherence Tomography Angiography Maps with a Deep Learning Model	https://iovs.arvojournals.org/article.aspx?articleid=2782927	The Association for Research in Vision and Ophthalmology Annual Meeting	Poster	Abstract
Nagamine et al.	2022	Abstract WMP67: Evaluation Of The Implementation Of An Ai Tool For Large Vessel Occlusion: Impact On Radiologists' Workflow And Patient Outcomes	10.1161/str.53.suppl_1.WMP67	International Stroke Conference	Poster	Abstract
Najac et al.	2022	Follow Up Metrics in Primary Care Clinics after Implementation of an Artificial Intelligence Assisted Telemedicine Screening Program for Diabetic Retinopathy	https://iovs.arvojournals.org/article.aspx?articleid=2780621	The Association for Research in Vision and Ophthalmology Annual Meeting	Poster	Abstract
Nakamura et al.	2019	A clinically applicable deep learning model for segmentation in the prostate region	10.1016/S0167-8140(19)32462-4	The European SocieTy for Radiotherapy and Oncology	Poster	Abstract
Ng et al.	2022	Safe and effective integration of AI as supporting reader in double reading breast cancer screening	10.1186/s13244-022-01337-x	European Congress of Radiology	Oral	Abstract
Nikola et al.	2023	AI-Assisted Imaging Implementation Within A Supraregional Mechanical Thrombectomy Network	10.1177/17474930221142512	UK Stroke Forum	NI	Abstract
Oberije et al.	2022	Post-market real-world data demonstrating use of an AI system as an extra reader to augment breast cancer detection without unnecessary recalls	10.1186/s13244-022-01337-x	European Congress of Radiology	Oral	Abstract
Parthasarathy et al.	2022	Performance of an Automated, Deep Learning-Based Tool to Screen for Age-Related Macular Degeneration (AMD)	https://iovs.arvojournals.org/article.aspx?articleid=2783124	The Association for Research in Vision and Ophthalmology Annual Meeting	Poster	Abstract
Parwani et al.	2020	Large Scale Deployment of Whole Slide Imaging for Anatomical Pathology Workflow Improvement and Primary Diagnosis and Consult Reviews	10.1038/s41374-020-0393-8	United States and Canadian Academy of Pathology Annual Meeting	Poster	Abstract
Pohlkamp et al.	2021	A Completely Digital Workflow for Differentials in Bone Marrow Cytomorphology Supported By Machine Learning Provides Promising Results in Object Detection	10.1182/blood-2021-152851	American Society of Hematology Annual Meeting	NI	Abstract
Raoux et al.	2021	Novel AI-Based Solution for Supporting Prostate Cancer Diagnosis Increases the Efficiency and Accuracy of Reporting in Clinical Routine	10.1038/s41374-021-00558-w	United States and Canadian Academy of Pathology Annual Meeting	NI	Abstract
Repici et al.	2020	876 Real-Time Computer Aided Diagnosis For Detection Of Colorectal Neoplasia At Colonoscopy	10.1016/j.gie.2020.03.604	Digestive Disease Week	Oral	Abstract
Rose et al.	2022	Img-11. A Computerised Clinical Decision Support System For Diagnosing Children's Brain Tumours Using Functional Imaging And Machine Learning	10.1093/neuonc/noac079.287	International Symposium on Pediatric Neuro-Oncology	NI	Abstract
Sandbank et al.	2022	Validation and Clinical Deployment of an AI-Based Algorithm for Detection of Gastric Adenocarcinoma and H. Pylori in Gastric Biopsies	https://ibex-ai.com/wp-content/	United States and Canadian Academy of Pathology Annual Meeting	Poster	Poster
Silva P et al.	2022	Automated machine learning (AutoML) model for diabetic retinopathy (DR) image classification from ultrawide field (UWF) retinal images	https://iovs.arvojournals.org/article.aspx?articleid=2782543	The Association for Research in Vision and Ophthalmology Annual Meeting	Poster	Abstract

Sukmanov et al.	2022	Successful deployment of an AI solution for prostate biopsies	10.1007/s00428-022-03379-4	European Congress of Pathology	Oral	Abstract
Takaishi	2022	The Use Of Artificial Intelligence System To Detect Lung Nodules On Ct Scan In Real Clinical Settings	10.1016/j.chest.2021.12.349	CHEST Congress	NI	Abstract
Taylor et al.	2022	MO012: Development of an Accurate Automated Segmentation Algorithm to Measure Total Kidney Volume in ADPKD Suitable for Clinical Application (The Cystvas Study)	10.1093/ndt/gfac061.007	European Renal Association Congress	Oral	Abstract
Ting et al.	2020	Artificial Intelligence-Assisted Diabetic Retinopathy Screening Program: A 5-year Bench to Bedside Translational Study	https://iovs.arvojournals.org/article.aspx?articleid=2767329	The Association for Research in Vision and Ophthalmology Annual Meeting	NI	Abstract
Wismüller & Stockmaster	2020	A Prospective Randomized Clinical Trial for Measuring Radiology Study Reporting Time on Artificial Intelligence-Based Detection of Intracranial Hemorrhage in Emergent Care Head CT	10.48550/ARXIV.2002.12515	The International Society for Optics und Photonics Medical Imaging Conference	Oral	Conference Paper
Yeo et al.	2022	A deep learning algorithm for the automatic detection of intracranial haemorrhage on CT head imaging	10.1186/s13244-022-01337-x	European Congress of Radiology	Oral	Abstract
Yi et al.	2020	Identification of Intracranial Hemorrhage Using an Original Artificial Intelligence System	10.1111/acem.13961	Society for Academic Emergency Medicine Annual Meeting	NI	Abstract
Younis et al.	2022	Integrating artificial Intelligence algorithms with DICOM structured reporting into clinical workflows	10.1186/s13244-022-01337-x	European Congress of Radiology	Oral	Abstract
Zia et al.	2021	Clinical use of Aidoc "Always-On AI": Does it help increaseradiologists' efficiency and improve patient care?	10.1111/1754-9485.13298	The Royal Australian and New Zealand College of Radiologists Annual Scientific Meeting	Oral	Abstract

Notes: NI = No information

SUPPLEMENTARY FIGURE 8: AMSTAR-2 CHECKLIST

The AMSTAR-2 (A MeaSurement Tool to Assess Systematic Reviews) critical appraisal tool for systematic reviews of randomized and/or non-randomized healthcare intervention studies was used to assess the quality of our review.

AMSTAR 2

1. Did the research questions and inclusion criteria for the review include the components of PICO?

For Yes:
- [x] Population
- [x] Intervention
- [x] Comparator group
- [x] Outcome

Optional (recommended)
- [ ] Timeframe for follow-up

- [x] Yes
- [ ] No

2. Did the report of the review contain an explicit statement that the review methods were established prior to the conduct of the review and did the report justify any significant deviations from the protocol?

For Partial Yes:
The authors state that they had a written protocol or guide that included ALL the following:
- [x] review question(s)
- [x] a search strategy
- [x] inclusion/exclusion criteria
- [x] a risk of bias assessment

For Yes:
As for partial yes, plus the protocol should be registered and should also have specified:
- [x] a meta-analysis/synthesis plan, if appropriate, *and*
- [x] a plan for investigating causes of heterogeneity
- [x] justification for any deviations from the protocol

- [x] Yes
- [ ] Partial Yes
- [ ] No

3. Did the review authors explain their selection of the study designs for inclusion in the review?

For Yes, the review should satisfy ONE of the following:
- [ ] *Explanation for* including only RCTs
- [ ] OR *Explanation for* including only NRSI
- [x] OR *Explanation for* including both RCTs and NRSI

- [x] Yes
- [ ] No

4. Did the review authors use a comprehensive literature search strategy?

For Partial Yes (all the following):
- [x] searched at least 2 databases (relevant to research question)
- [x] provided key word and/or search strategy
- [x] justified publication restrictions (eg, language)

For Yes, should also have (all the following):
- [x] searched the reference lists/bibliographies of included studies
- [x] searched trial/study registries
- [ ] included/consulted content experts in the field
- [ ] where relevant, searched for grey literature
- [x] conducted search within 24 months of completion of the review

- [ ] Yes
- [x] Partial Yes
- [ ] No

5. Did the review authors perform study selection in duplicate?

For Yes, either ONE of the following:
- [x] at least two reviewers independently agreed on selection of eligible studies and achieved consensus on which studies to include
- [ ] OR two reviewers selected a sample of eligible studies and achieved good agreement (at least 80 per cent), with the remainder selected by one reviewer

- [x] Yes
- [ ] No

6. Did the review authors perform data extraction in duplicate?

For Yes, either ONE of the following:
- [x] at least two reviewers achieved consensus on which data to extract

- [x] Yes

from included studies<br>☐ OR two reviewers extracted data from a sample of eligible studies and achieved good agreement (at least 80 per cent), with the remainder extracted by one reviewer		☐ No

7. Did the review authors provide a list of excluded studies and justify the exclusions?

For Partial Yes:	For Yes, must also have:	
✔ provided a list of all potentially relevant studies that were read in full text form but excluded from the review	✔ Justified the exclusion from the review of each potentially relevant study	✔ Yes<br>☐ Partial Yes<br>☐ No

8. Did the review authors describe the included studies in adequate detail?

For Partial Yes (ALL the following):	For Yes, should also have ALL the following:	
✔ described populations<br>✔ described interventions<br>✔ described comparators<br>✔ described outcomes<br>✔ described research designs	✔ described population in detail<br>✔ described intervention and comparator in detail (including doses where relevant)<br>✔ described study's setting<br>☐ timeframe for follow-up N.A.	✔ Yes<br>☐ Partial Yes<br>☐ No

9. Did the review authors use a satisfactory technique for assessing the risk of bias (RoB) in individual studies that were included in the review?

**RCTs**<br>For Partial Yes, must have assessed RoB from	For Yes, must also have assessed RoB from:	
✔ unconcealed allocation, *and*<br>✔ lack of blinding of patients and assessors when assessing outcomes (unnecessary for objective outcomes such as all cause mortality)	✔ allocation sequence that was not truly random, *and*<br>✔ selection of the reported result from among multiple measurements or analyses of a specified outcome	✔ Yes<br>☐ Partial Yes<br>☐ No<br>☐ Includes only NRSI
**NRSI**<br>For Partial Yes, must have assessed RoB:	For Yes, must also have assessed RoB:	
✔ from confounding, *and*<br>✔ from selection bias	✔ methods used to ascertain exposures and outcomes, *and*<br>✔ selection of the reported result from among multiple measurements or analyses of a specified outcome	✔ Yes<br>☐ Partial Yes<br>☐ No<br>☐ Includes only RCTs

10. Did the review authors report on the sources of funding for the studies included in the review?

For Yes	
✔ Must have reported on the sources of funding for individual studies included in the review. Note: Reporting that the reviewers looked for this information but it was not reported by study authors also qualifies	✔ Yes<br>☐ No

11. If meta-analysis was performed did the review authors use appropriate methods for statistical combination of results?

**RCTs**<br>For Yes:	
✔ The authors justified combining the data in a meta-analysis<br>✔ AND they used an appropriate weighted technique to combine study results and adjusted for heterogeneity if present	✔ Yes<br>☐ No<br>☐ No meta-analysis

✓ AND investigated the causes of any heterogeneity	conducted
**For NRSI**<br>For Yes:<br>✓ The authors justified combining the data in a meta-analysis<br>✓ AND they used an appropriate weighted technique to combine study results, adjusting for heterogeneity if present<br>✓ AND they statistically combined effect estimates from NRSI that were adjusted for confounding, rather than combining raw data, or justified combining raw data when adjusted effect estimates were not available<br>☐ AND they reported separate summary estimates for RCTs and NRSI separately when both were included in the review	☐ Yes<br>✓ No<br>☐ No meta-analysis conducted

12. If meta-analysis was performed, did the review authors assess the potential impact of RoB in individual studies on the results of the meta-analysis or other evidence synthesis?

For Yes:<br>☐ included only low risk of bias RCTs<br>✓ OR, if the pooled estimate was based on RCTs and/or NRSI at variable RoB, the authors performed analyses to investigate possible impact of RoB on summary estimates of effect	✓ Yes<br>☐ No<br>☐ No meta-analysis conducted

13. Did the review authors account for RoB in individual studies when interpreting/discussing the results of the review?

For Yes:<br>☐ included only low risk of bias RCTs<br>✓ OR, if RCTs with moderate or high RoB, or NRSI were included the review provided a discussion of the likely impact of RoB on the results	✓ Yes<br>☐ No

14. Did the review authors provide a satisfactory explanation for, and discussion of, any heterogeneity observed in the results of the review?

For Yes:<br>☐ There was no significant heterogeneity in the results<br>✓ OR if heterogeneity was present the authors performed an investigation of sources of any heterogeneity in the results and discussed the impact of this on the results of the review	✓ Yes<br>☐ No

15. If they performed quantitative synthesis did the review authors carry out an adequate investigation of publication bias (small study bias) and discuss its likely impact on the results of the review?

For Yes:<br>✓ performed graphical or statistical tests for publication bias and discussed the likelihood and magnitude of impact of publication bias	✓ Yes<br>☐ No<br>☐ No meta-analysis conducted

16. Did the review authors report any potential sources of conflict of interest, including any funding they received for conducting the review?

For Yes:<br>✓ The authors reported no competing interests OR<br>☐ The authors described their funding sources and how they managed potential conflicts of interest	✓ Yes<br>☐ No

SUPPLEMENTARY TABLE 3: PRISMA REPORTING CHECKLIST

Section and Topic	Item #	Checklist item	Location where item is reported
**TITLE**			
Title	1	Identify the report as a systematic review.	1
**ABSTRACT**			
Abstract	2	See the PRISMA 2020 for Abstracts checklist.	2
**INTRODUCTION**			
Rationale	3	Describe the rationale for the review in the context of existing knowledge.	3
Objectives	4	Provide an explicit statement of the objective(s) or question(s) the review addresses.	3
**METHODS**			
Eligibility criteria	5	Specify the inclusion and exclusion criteria for the review and how studies were grouped for the syntheses.	14-16
Information sources	6	Specify all databases, registers, websites, organisations, reference lists and other sources searched or consulted to identify studies. Specify the date when each source was last searched or consulted.	14-15, 17-18
Search strategy	7	Present the full search strategies for all databases, registers and websites, including any filters and limits used.	Supplementary Note 1-2
Selection process	8	Specify the methods used to decide whether a study met the inclusion criteria of the review, including how many reviewers screened each record and each report retrieved, whether they worked independently, and if applicable, details of automation tools used in the process.	16
Data collection process	9	Specify the methods used to collect data from reports, including how many reviewers collected data from each report, whether they worked independently, any processes for obtaining or confirming data from study investigators, and if applicable, details of automation tools used in the process.	16
Data items	10a	List and define all outcomes for which data were sought. Specify whether all results that were compatible with each outcome domain in each study were sought (e.g. for all measures, time points, analyses), and if not, the methods used to decide which results to collect.	15-16
	10b	List and define all other variables for which data were sought (e.g. participant and intervention characteristics, funding sources). Describe any assumptions made about any missing or unclear information.	15
Study risk of bias assessment	11	Specify the methods used to assess risk of bias in the included studies, including details of the tool(s) used, how many reviewers assessed each study and whether they worked independently, and if applicable, details of automation tools used in the process.	16-17
Effect measures	12	Specify for each outcome the effect measure(s) (e.g. risk ratio, mean difference) used in the synthesis or presentation of results.	17
Synthesis methods	13a	Describe the processes used to decide which studies were eligible for each synthesis (e.g. tabulating the study intervention characteristics and comparing against the planned groups for each synthesis (item #5)).	17
	13b	Describe any methods required to prepare the data for presentation or synthesis, such as handling of missing summary statistics, or data conversions.	17
	13c	Describe any methods used to tabulate or visually display results of individual studies and syntheses.	17

Section and Topic	Item #	Checklist item	Location where item is reported
	13d	Describe any methods used to synthesize results and provide a rationale for the choice(s). If meta-analysis was performed, describe the model(s), method(s) to identify the presence and extent of statistical heterogeneity, and software package(s) used.	17
	13e	Describe any methods used to explore possible causes of heterogeneity among study results (e.g. subgroup analysis, meta-regression).	17
	13f	Describe any sensitivity analyses conducted to assess robustness of the synthesized results.	17-18
Reporting bias assessment	14	Describe any methods used to assess risk of bias due to missing results in a synthesis (arising from reporting biases).	17-18
Certainty assessment	15	Describe any methods used to assess certainty (or confidence) in the body of evidence for an outcome.	18
**RESULTS**			
Study selection	16a	Describe the results of the search and selection process, from the number of records identified in the search to the number of studies included in the review, ideally using a flow diagram.	4, Figure 1
	16b	Cite studies that might appear to meet the inclusion criteria, but which were excluded, and explain why they were excluded.	Supplementary Table 1-2
Study characteristics	17	Cite each included study and present its characteristics.	Table 1
Risk of bias in studies	18	Present assessments of risk of bias for each included study.	Supplementary Figure 1-2
Results of individual studies	19	For all outcomes, present, for each study: (a) summary statistics for each group (where appropriate) and (b) an effect estimate and its precision (e.g. confidence/credible interval), ideally using structured tables or plots.	Table 4
Results of syntheses	20a	For each synthesis, briefly summarise the characteristics and risk of bias among contributing studies.	4-6
	20b	Present results of all statistical syntheses conducted. If meta-analysis was done, present for each the summary estimate and its precision (e.g. confidence/credible interval) and measures of statistical heterogeneity. If comparing groups, describe the direction of the effect.	7-8, Figure 3
	20c	Present results of all investigations of possible causes of heterogeneity among study results.	7-8
	20d	Present results of all sensitivity analyses conducted to assess the robustness of the synthesized results.	9-10
Reporting biases	21	Present assessments of risk of bias due to missing results (arising from reporting biases) for each synthesis assessed.	Supplementary Figure 5-7
Certainty of evidence	22	Present assessments of certainty (or confidence) in the body of evidence for each outcome assessed.	Supplementary Figure 3-4
**DISCUSSION**			
Discussion	23a	Provide a general interpretation of the results in the context of other evidence.	10-12

Section and Topic	Item #	Checklist item	Location where item is reported
	23b	Discuss any limitations of the evidence included in the review.	13
	23c	Discuss any limitations of the review processes used.	13
	23d	Discuss implications of the results for practice, policy, and future research.	14
**OTHER INFORMATION**			
Registration and protocol	24a	Provide registration information for the review, including register name and registration number, or state that the review was not registered.	2, 14
	24b	Indicate where the review protocol can be accessed, or state that a protocol was not prepared.	14
	24c	Describe and explain any amendments to information provided at registration or in the protocol.	16-17
Support	25	Describe sources of financial or non-financial support for the review, and the role of the funders or sponsors in the review.	18
Competing interests	26	Declare any competing interests of review authors.	18
Availability of data, code and other materials	27	Report which of the following are publicly available and where they can be found: template data collection forms; data extracted from included studies; data used for all analyses; analytic code; any other materials used in the review.	18

From: Page MJ, McKenzie JE, Bossuyt PM, Boutron I, Hoffmann TC, Mulrow CD, et al. The PRISMA 2020 statement: an updated guideline for reporting systematic reviews. BMJ 2021;372:n71. doi: 10.1136/bmj.n71

SUPPLEMENTARY TABLE 4: PICO STATEMENT

	CONNECTOR	SEARCH TERM
**POPULATION**		"hospital" OR "clinic" OR "healthcare" OR "health care delivery" OR "clinical care" OR "medical" OR physician* OR clinician* OR doctor* OR nurse* OR "health care professional" OR "patient care" OR patient* OR surg* OR "oncology" OR "radiology" OR "health information"
**INTERVENTION**	AND	"artificial intelligence" OR "machine intelligence" OR "machine learning" OR "deep learning" OR "neural network" OR "natural language processing" OR "AI " OR "automated image recognition" OR "decision-support" OR "AI application*"
	AND	"adoption" OR "deploy*" OR "implementation" OR "integration"
	AND	diagnos* OR "Magnetic Resonance Imaging" OR MRI OR "computer tomography" OR imag* OR detect* OR "data interpretation" OR "information system*" OR "health information technology*" OR "health IT*" OR "medical informatics" OR "electronic health record*" OR "medical record*" OR "patient data"
**OUTCOMES**	AND	"workload" OR "work reduction" OR load* OR "cognitive load" OR demand* OR time* OR stress* OR "satisfaction" OR "usability" OR "workflow" OR efficienc* OR "work system" OR "work adaptation" OR "turnaround" OR "clinician outcome" OR "performance"

Source: Wenderott, K., Gambashidze, N., & Weigl, M. (2022). Integration of Artificial Intelligence Into Sociotechnical Work Systems—Effects of Artificial Intelligence Solutions in Medical Imaging on Clinical Efficiency: Protocol for a Systematic Literature Review. *JMIR Research Protocols*, *11*(12), e40485. https://doi.org/10.2196/40485

SUPPLEMENTARY NOTE 1: DETAILED SEARCH STRATEGY

Pubmed, Web of Science, CENTRAL

Filter: 2000-[date}

("hospital" OR "clinic" OR "healthcare" OR "health care delivery" OR "clinical care" OR "medical" OR physician* OR clinician* OR doctor* OR nurse* OR "health care professional" OR "patient care" OR patient* OR surg* OR "oncology" OR "radiology" OR "health information")

AND ("artificial intelligence" OR "machine intelligence" OR "machine learning" OR "deep learning" OR "neural network" OR "natural language processing" OR "AI " OR "automated image recognition" OR "decision-support" OR "AI application*")

AND ("adoption" OR "deploy*" OR "implementation" OR "integration")

AND ("workload" OR "work reduction" OR load* OR "cognitive load" OR demand* OR time* OR stress* OR "satisfaction" OR "usability" OR "workflow" OR efficienc* OR "work system" OR "work adaptation" OR "turnaround" OR "clinician outcome" OR "performance")

AND (diagnos*[mh:noexp] OR "Magnetic Resonance Imaging" OR MRI[mh:noexp] OR "computer tomography" OR imag*[mh:noexp] OR detect* OR "data interpretation" OR "information system*" OR "health information technology*" OR "health IT*" OR "medical informatics" OR "electronic health record*" OR "medical record*" OR "patient data")

Embase

Filter: 2000-[date]

(hospital OR clinic OR healthcare OR "health care delivery" OR "clinical care" OR medical OR physician* OR clinician* OR doctor* OR nurse* OR "health care professional" OR "patient care" OR patient* OR surg* OR oncology OR radiology OR "health information")

AND ("artificial intelligence" OR "machine intelligence" OR "machine learning" OR "deep learning" OR "neural network" OR "natural language processing" OR AI OR "automated image recognition" OR decision-support OR "AI application*")

AND (adoption OR deploy* OR implementation OR integration)

AND (workload OR "work reduction" OR load* OR "cognitive load" OR demand* OR time* OR stress* OR satisfaction OR usability OR workflow OR efficienc* OR "work system" OR "work adaptation" OR turnaround OR "clinician outcome" OR performance)

AND (diagnos*/ OR "Magnetic Resonance Imaging" OR MRI/ OR "computer tomography" OR imag*/ OR detect* OR "data interpretation" OR "information system*" OR "health information technology*" OR "health IT*" OR "medical informatics" OR "electronic health record*" OR "medical record*" OR "patient data")

Psycinfo (Ebsco)

Note: Did not accept capital letters, therefore small changes in search term

Filter: 2000-[date]

(hospital OR clinic OR healthcareOR "health care delivery"OR "clinical care"OR medicalOR physician*OR clinician*OR doctor*OR nurse*OR "health care professional"OR "patient care"OR patient*OR surg*OR oncologyOR radiologyOR "health information")

AND ("artificial intelligence"OR "machine intelligence" OR "machine learning"OR "deep learning"OR "neural network"OR "natural language processing"OR “AI” OR "automated image recognition"OR decision-supportOR "AI application*")

AND (adoptionOR deploy*OR implementationOR integration)

AND (workloadOR "work reduction"OR load*OR "cognitive load"OR demand*OR time*OR stress*OR satisfactionOR usabilityOR workflowOR efficienc*OR "work system"OR "work adaptation"OR turnaroundOR "clinician outcome"OR performance)

AND (“diagnos*” OR "Magnetic Resonance Imaging"OR “MRI” OR "computer tomography"OR “imag*” OR detect*OR "data interpretation"OR "information system*"OR "health information technology*"OR "health IT*"OR "medical informatics"OR "electronic health record*"OR "medical record*"OR "patient data")

IEEE Xplore

Note: Problems with more than 8 wildcards, therefore changes in search term

Filter: Journals, Magazines, Early Access Articles; 2000-[date]

(hospital OR clinic OR healthcare OR "health care delivery" OR "clinical care" OR medical OR physician OR clinician OR doctor OR nurse OR physicians OR clinicians OR doctors OR nurses OR "health care professional" OR "patient care" OR patient OR patients OR surg* OR oncology OR radiology OR "health information")

AND ("artificial intelligence" OR "machine intelligence" OR "machine learning" OR "deep learning" OR "neural network" OR "natural language processing" OR “AI” OR "automated image recognition" OR decision-support OR "AI application" OR "AI applications")

AND (adoption OR deploy* OR implementation OR integration)

AND (workload OR "work reduction" OR load OR loads OR "cognitive load" OR demand OR time* OR stress OR stressful OR stressing OR satisfaction OR usability OR workflow OR efficienc* OR "work system" OR "work adaptation" OR turnaround OR "clinician outcome" OR performance)

AND (“diagnos*” OR "Magnetic Resonance Imaging" OR “MRI” OR "computer tomography" OR “imag*” OR detect* OR "data interpretation" OR "information system" OR "health information technology" OR "health information technologies” OR "health IT" OR "medical informatics" OR "electronic health record" OR "medical record" OR "electronic health records" OR "medical records" OR "patient data")

SUPPLEMENTARY NOTE 2: ADDITIONAL SEARCHES TO ASSESS POTENTIAL SELECTION BIAS

dblp computer science bibliography

Note: More than two search terms resulted in a low number or no findings, therefore we conducted six very general searches. For the publication date, we filtered by using MS Excel on the exported records.

NUMBER	SEARCH TERM
1	Artificial intelligence healthcare
2	Artificial intelligence hospital
3	Artificial intelligence medical imaging
4	Machine learning healthcare
5	Machine learning hospital
6	Machine learning medical imaging

IEEE Xplore

Filter: Conferences 2000-2023

Note: For the exact publication date, we filtered the column “Date added to IEEE Xplore” by using MS Excel on the exported records.

(hospital OR clinic OR healthcare OR "health care delivery" OR "clinical care" OR medical OR physician OR clinician OR doctor OR nurse OR physicians OR clinicians OR doctors OR nurses OR "health care professional" OR "patient care" OR patient OR patients OR surg* OR oncology OR radiology OR "health information")

AND ("artificial intelligence" OR "machine intelligence" OR "machine learning" OR "deep learning" OR "neural network" OR "natural language processing" OR “AI” OR "automated image recognition" OR decision-support OR "AI application" OR "AI applications")

AND (adoption OR deploy* OR implementation OR integration)

AND (workload OR "work reduction" OR load OR loads OR "cognitive load" OR demand OR time* OR stress OR stressful OR stressing OR satisfaction OR usability OR workflow OR efficienc* OR "work system" OR "work adaptation" OR turnaround OR "clinician outcome" OR performance)

AND (“diagnos*” OR "Magnetic Resonance Imaging" OR “MRI” OR "computer tomography" OR “imag*” OR detect* OR "data interpretation" OR "information system" OR "health information technology" OR "health information technologies” OR "health IT" OR "medical informatics" OR "electronic health record" OR "medical record" OR "electronic health records" OR "medical records" OR "patient data")

Medical Image Computing and Computer Assisted Intervention (MICCAI)

The proceedings of the MICCAI conference are not searchable on Springer Link, therefore we retrieved the studies from https://github.com/JunMa11/MICCAI-OpenSourcePapers, drawing upon the Proceedings 2020 and the list of accepted Papers from 2021 and 2022.

Hawaii International Conference on System Sciences (HICSS)

Note: Search via AIS Library, which did not take the complete search strings therefore we adapted it.

Filter: 2020-2022

(hospital OR clinic OR healthcare OR "health care delivery" OR "clinical care" OR medical OR physician OR clinician OR doctor OR nurse OR physicians OR clinicians OR doctors OR nurses OR "health care professional" OR "patient care" OR patient OR patients OR surg* OR oncology OR radiology OR "health information")

AND ("artificial intelligence" OR "machine intelligence" OR "machine learning" OR "deep learning" OR "neural network" OR "natural language processing" OR “AI” OR "automated image recognition" OR decision-support OR "AI application" OR "AI applications")

AND (adoption OR deploy* OR implementation OR integration)

AND (workload OR "work reduction" OR load OR loads OR "cognitive load" OR demand OR time* OR stress OR stressful OR stressing OR satisfaction OR usability OR workflow OR efficienc* OR "work system" OR "work adaptation" OR turnaround OR "clinician outcome" OR performance)
